# Sesquiterpene Lactones from *Cotula cinerea* with Antibiotic Activity against Clinical Isolates of *Enterococcus faecalis*

**DOI:** 10.3390/antibiotics10070819

**Published:** 2021-07-06

**Authors:** Alessio Cimmino, Emanuela Roscetto, Marco Masi, Angela Tuzi, Imene Radjai, Chakali Gahdab, Rossella Paolillo, Amedeo Guarino, Maria Rosaria Catania, Antonio Evidente

**Affiliations:** 1 Department of Chemical Sciences, University of Naples Federico II, Complesso Universitario Monte Sant’Angelo, via Cintia 4, 80126 Napoli, Italy; marco.masi@unina.it (M.M.); angela.tuzi@unina.it (A.T.); evidente@unina.it (A.E.); 2 Department of Molecular Medicine and Medical Biotechnologies, University of Naples Federico II, via Pansini 5, 80131 Napoli, Italy; rossella.paolillo@unina.it (R.P.); mariarosaria.catania@unina.it (M.R.C.); 3 Ecole Nationale Supérieure Agronomique, Département de Zoologie Agricole et Forestière, Belfort, El-Harrach, Algers 16200, Algeria; imerad50@gmail.com (I.R.); chakali_gahdab@yahoo.fr (C.G.); 4 Department of Public Health, Section of Orthopaedic Surgery, University of Naples Federico II, via Pansini 5, 80131 Napoli, Italy; amedeo.guarino1@gmail.com

**Keywords:** *Cotula cinerea*, sesquiterpenes, germacrenolides, guaianolides, *Enterococcus faecalis*, antibiotic activity, antibiofilm

## Abstract

*Cotula cinerea*, belonging to the tribe Anthemideae, is a plant widespread in the Southern hemisphere. It is frequently used in folk medicine in North African countries for several of its medical properties, shown by its extracts and essential oils. The dichloromethane extract obtained from its aerial parts demonstrated antibiotic activity against *Enterococcus faecalis* and was fractionated by bioguided purification procedures affording five main sesquiterpene lactones. They were identified by spectroscopic methods (NMR and ESIMS data) as guaiantrienolides, i.e., 6-acetoxy-1β-,6-acetoxy-1α-, and 6-acetoxy-10-β-hydroxyguaiantrienolide (**1**–**3**), and germacrenolides, i.e., haagenolide and 1,10-epoxyhaagenolide (**4** and **5**). The absolute configuration was assigned by applying the advanced Mosher’s method to haagenolide and by X-ray diffraction analysis to 1,10-epoxyhaagenolide. The specific antibiotic and antibiofilm activities were tested toward the clinical isolates of *Enterococcus faecalis*. The results showed that compounds **3**–**5** have antibacterial activity against all the strains of *E. faecalis*, while compound **2** exhibited activity only toward some strains. Compound **1** did not show this activity but had only antibiofilm properties. Thus, these metabolites have potential as new antibiotics and antibiofilm against drug resistant opportunistic pathogens.

## 1. Introduction

There has been remarkable interest in the last twenty years in the discovery of natural substances as a potential reservoir of innovative therapeutic solutions for human health, with the prospect of integrating and sometimes replacing conventional drugs. In particular, there is a growing interest in the study of natural substances extracted from plants to counter the galloping phenomenon of antibiotic resistance, i.e., the development and spread of microorganisms resistant to the drugs used in anti-infective therapies [1,2,3,4]. The misuse and abuse of antimicrobials in humans and animals have accelerated the growing phenomenon of antimicrobial resistance worldwide. Over the last few decades, antibiotic resistance (AR) has become a global threat to health systems and public health around the world. If it cannot be effectively counteracted, it is estimated that by 2050 the spread of multidrug-resistant strains will hamper the control of many infectious diseases, undermining the achievements of modern medicine [5].

Today, two main problems affect the effectiveness of antibiotics. The first is that following the introduction of a new antibiotic, resistance to it will arise sooner or later; the second is the growing gap between the increase in antimicrobial resistance and the development of new compounds. This means that the pace of discovery and development of new antibiotics is slower than the emergence and spread of resistance mechanisms among bacteria, which are able to respond rapidly to selective pressures and pass resistance genes to the offspring.

About 80% of microbial infections are associated with the formation of biofilm, a film formed by bacteria enclosed in a self-produced extracellular polymeric matrix that adheres to biotic and abiotic surfaces such as those of implantable materials (prostheses, cardiac devices or dental implants) [6,7,8,9]. The antimicrobial resistance of biofilms is not genotypic; instead, it occurs due to the multicellular strategies and/or the ability of individual cells, within the biofilm, to differentiate in a phenotypic state tolerant of the action of antimicrobials. Several studies have shown that living bacteria in sessile form are much less susceptible to antibiotics than living bacteria in planktonic form because they are protected by the matrix of the biofilm. In fact, this represents a barrier for antimicrobial agents, blocking or delaying their diffusion, or interacting chemically with the antibiotic [10,11]. Another mechanism responsible for the resistance of biofilms to antimicrobial agents is linked to the timing of cell growth. Cells that live in sessile form have a much slower growth time than planktonic cells and can consequently take on antimicrobial agents more slowly [12]. The fight against AR has therefore been a challenge for doctors and researchers all over the world for more than twenty years.

One of the sources of new antibiotics that have original carbon skeleton to overcome AR are plants. They produce a plethora of bioactive secondary metabolites as thousands of alkaloids, polyketides, phenylpropanoids, phenols, quinones, terpenes, flavanoids, etc. Several of these metabolites are used as defense against pathogens and are involved in electron transport chains, mitochondria function, and membrane integrity [13,14]. Plant metabolites could have different applications in agriculture as biopesticides [15], but they also have several applications in medicine for new drug discovery [16]. Thus, this kingdom has a particular niche, including native plants, many of which were used in folk medicine.

Among them there is *Cotula cinerea* L., syn. *Brocchia cinerea* Del. (Asteraceae), belonging to *Cotula* L., the largest genus in the Anthemideae tribe, which is widespread in the Southern hemisphere, including about 80 species [17]. The Anthemideae was grouped into seven provisional subtribes, and *Cotula* was placed in the group “Cotuleae” by Lloyd (1972) [18]. *C. cinerea*, commonly known as “Al gartoufa”, is used in Morocco as an anti-inflammatory, analgesic, antiseptic treatment and for the treatment of stomachache [19]. In Algerian Sahara, its hydroethanolic and infusion extracts were used for their antioxidant, anti-inflammatory, cytotoxic, and antimicrobial properties [20]. Recently, the antioxidant, antidiabetic, and antilipidemic effects of the aqueous extracts of its aerial parts were assessed [21].

A phytochemical investigation was carried out in 1976 on *C. cinerea* roots and different flavonoids as kaempferitin, quercetrin, quercetin, and kaempferol were isolated [22]. Successively, from another population of the same plant, luteolin and its 7-*O*-β-d-glucoside, 7-*O*-β-d-diglucoside and 6-hydroxy-7-*O*-β-d-glucoside, apigenin, 7-*O*-L-rhamnoside, 6-C-arabinosyl-8-C-glucosylapigenin, and isochaftoside were isolated as the primary flavonoids as well as to lesser extent the 3-*O*-β-d-glucoside, 3-*O*-β-d-galactoside, 7-3-*O*-β-d-glucoside of quercetin, and 5,3′,4′-trihydroxy 3,6,7-trimethoxyflavone [23]. In addition, a characteristic diacetylenic spiroketal enol-ether and several sesquiterpene lactones, three of them being glucosylated, were also isolated from the plant aerial parts [24]. From the same extract, new isofraxidin-derived sesquiterpene ethers and the new 8-farnesylscopoletin were isolated together with known spiroketal-enol ether polyenes and sesquiterpene coumarin ethers [25], and these metabolites seemed to have chemotaxonomic value.

The biological activities of aqueous and organic extracts (ethyl ether, ethyl acetate, and *n*-butanol) obtained from this plant were investigated as the antibacterial activity against *Pseudomonas* and *Bacillus* spp. [19], as the larvicidal activity against *Anopheles labranchiae* mosquito larvae [26], as the antipyretic activity in rats [27], and as the antifungal activity against *Fusarium graminearum* and *Fusarium sporotrichioides* [28]. Furthermore, the antioxidant, anti-inflammatory, cytotoxic, and antimicrobial activities of its hydroethanolic and infusion extracts were also described [25]. Finally, it was also used as a bioherbicide for the biological control of the noxious weed *Melilotus indicus* L. [29].

However, up to now any report that exists has described biological activities of the metabolites isolated from this plant, and some of them are only partially chemical characterized. In addition, the organic plant extracts showed variability in the quality and quantity of their components. The differences in chemical profile do not surprise, and as for other plants they could be assigned to several factors, such as the geographical origin, the inter- and intraspecific genetic variability, and the plant vegetative stage.

In our study, organic extracts from *Cotula cinerea* isolated in Algerian Sahara are tested against both Gram-positive and Gram-negative bacteria. The preliminary antibiotic activity of the CH_2_Cl_2_ plant extract prompts a complete chemical and biological characterization of the secondary metabolites. Thus, this manuscript reports the complete spectroscopic properties, the absolute stereochemistry of five sesquiterpene lactones belonging to guaianolides and germacrenolides subgroups, and their specific antibiotic and antibiofilm activity against the clinical and reference isolates of *Enterococcus faecalis*.

## 2. Results and Discussion

The organic extracts of *C. cinerea* obtained using different solvents (in succession *n*-hexane, CH_2_Cl_2_ and EtOAc) as detailed in Section 3 were assayed at a single high concentration on *Staphylococcus aureus* ATCC 29231, *Enterococcus faecalis* ATCC 29212, *Pseudomonas aeruginosa* ATCC 27853, *Acinetobacter baumannii* ATCC 747 by standard broth microdilution assay to test the bacterial growth inhibition. Among them, only the CH_2_Cl_2_ extract showed an antibacterial activity (Table 1), inhibiting in particular only the growth of Gram-positive test strains with 49% ± 2.7 of inhibition for *S. aureus* and 90% ± 1.5 for *E. faecalis*.

Markouk et al. (1999) [19] showed that the *n*-butanol extract of *C. cinerea* was active against phytopathogenic strains of *Pseudomonas* and *Bacillus*. The *C. cinerea n*-butanol extract was found to be active also against clinical isolates of *Klebsiella pneumoniae*, *Pseudomonas aeruginosa*, and *S. aureus* [30]. Ghouti et al. (2018) [20] tested a hydroethanolic extract and infusion of *C. cinerea* against both Gram-negative and Gram-positive bacteria, and the findings showed a higher activity toward Gram-positive ones.

The high efficacy showed by the CH_2_Cl_2_ plant extract in inhibiting the planktonic growth of *E. faecalis* appeared to be very interesting. *E. faecalis*, a commensal organism of human gastrointestinal tract, is recognized as an important nosocomial pathogen whose control is of paramount importance since this bacterium can cause bacteremia, urinary tract infections, endocarditis, intra-abdominal and pelvic infections, and burn and surgical site wound infections [31,32,33]. It has also become increasingly recognized as an important pathogen that causes infections of catheters and other implanted medical devices, including those that are orthopedic [34,35,36]. *E. faecalis* is in fact the most frequently identified species in enterococcal prosthetic joint infection (PJI) [37,38]. In dentistry, *E. faecalis* is particularly prevalent in root canals with a diagnosis of apical periodontitis and has been implicated as the main pathogen in persistent secondary endodontic infections [39,40]. The success of *E. faecalis* as a pathogen mostly linked to hospital-acquired infections (HAIs) is due to its intrinsic resistance to many antimicrobials and its capacity to acquire new genetic determinants of resistance to different classes of antibiotics. Furthermore, it is capable of forming a well-organized biofilm, which makes it more resistant to antibiotic killing and the action of human immune defenses, and it tolerates extreme environmental conditions (poor nutrient availability, low O_2_ potential, alkaline pH) [41]. Furthermore, it can induce hydroxyapatite precipitation in a mature biofilm to form a calcified biofilm [42].

**Figure 1:**
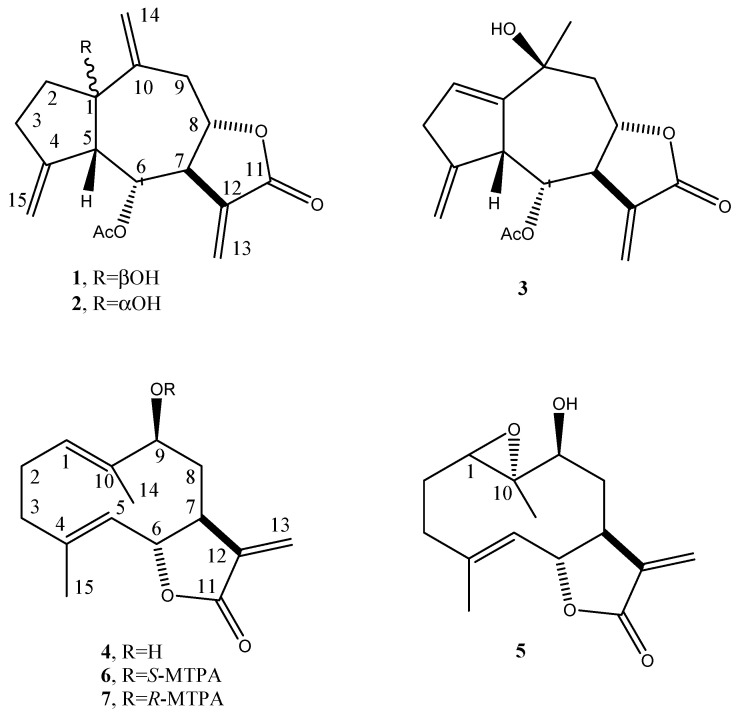
Structure of 6-acetoxy-1β-, 6-acetoxy-1α-, and 6-acetoxy-10-β-hydroguaiantrienolide, haagenolide, and 1,10-epoxyhaagenolide (**1**–**5**), and the diastereomeric *S*- and *R*-MTPA esters of haagenolide (**6** and **7**).

Thus, the active CH_2_Cl_2_ extract was fractionated by combined column and TLC on direct and reverse phase to afford five pure metabolites. The first investigation of their ^1^H NMR and ESI MS spectra showed that they are sesquiterpenoids belonging to different subgroups. They were identified as the guaianolides, i.e., 6-acetoxy-1β-, 6-acetoxy-1α- and 6-acetoxy-10-β-hydroxyguaiantrienolide (**1**–**3**, Figure 1), and the germacrenolides, i.e., haagenolide and 1,10-epoxyhaagenolide (**4** and **5**, Figure 1), by comparing their ^1^H NMR and MS data with those reported in literature, in particular for **1**–**3** with the data reported by Jakupovic et al. (1988) [43] when isolated from the same plant and for **4** and **5** with those reported when isolated, respectively, from the *Inula* species [44] and from *Inula heterolepis* [45].

However, the authors that were cited above only partially reported the ^1^H NMR data of the five sesquiterpenoids. Thus, by extensive use of 1D and 2D ^1^H and ^13^C NMR spectra (COSY, HSQC and HMBC), the chemical shifts to all the carbons and protons were assigned for the first time and reported in Table 2, Table 3, Table 4, Table 5 and Table 6.

The relative configuration of **1**–**5** were supported by correlations observed in their NOESY spectra (see Figures S6, S12, S18, S24 and S30).

The absolute configuration of haagenolide (**4**) was determined by applying the advanced Mosher’s method [46] to the hydroxylated methine (C-9). The haagenolide (**4**) was converted into the corresponding *S*-MTPA (**6**) and *R*-MTPA (**7**) monoesters at C-9 (Figure 1) by reaction with *R*-(−)-α-methoxy-α-trifluoromethyl-α-phenylacetyl (MTPA) and *S*-(+)-MTPA chloride, the spectroscopic data of which were consistent with the structure assigned to **4**.

**Figure 2:**
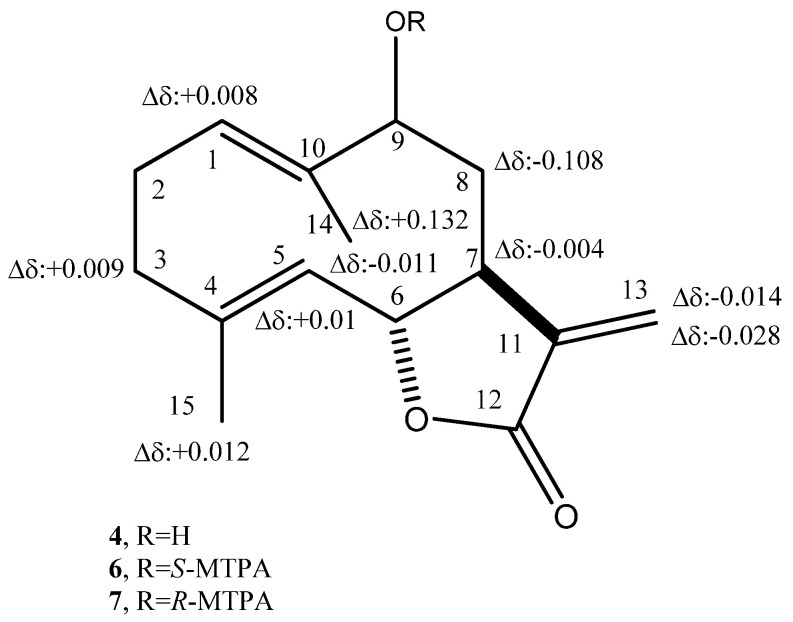
Structures of 9-*O*-*S*- (**6**) and 9-*O*-*R*-MTPA (**7**), esters of haagenolide (**4**), reporting the Δδ values obtained by comparison of each proton system.

The ^1^H NMR spectra of both **6** and **7** (Table 7) substantially differed from that of **4** for the downfield shift (Δδ 1.27 and 1.24, respectively) of H-9 and for the presence of the proton of benzene ring system and the singlet of the methoxy group at δ 7.508–7.384 and 7.532–7.367 and 3.519 and 3.557, respectively. Subtracting the chemical shifts of the protons (Table 6) of 9-*O*-*S*-MTPA of **4** (**7**) from those of 9-*O*-*R*-MTPA of **4** (**6**) esters, the Δδ (**7**–**6**) values for all of the protons were determined as reported in Figure 2. The positive Δδ values are located on the right-hand side and the negative values on the left-hand side of model A as reported previously [46]. This model allowed us to assign the *S* configuration to C-9 and consequently the *S* and *R* configuration to C-7 and C-6, respectively. Thus, **4** was formulated as (6*R*,7*S*,9*S*)-5-hydroxy-6,10-dimethyl-3-methylene-3*a*,4,5,8,9,11*a*-hexahydro-3*H*-cyclodeca[*b*]furan-2-one.

**Figure 3:**
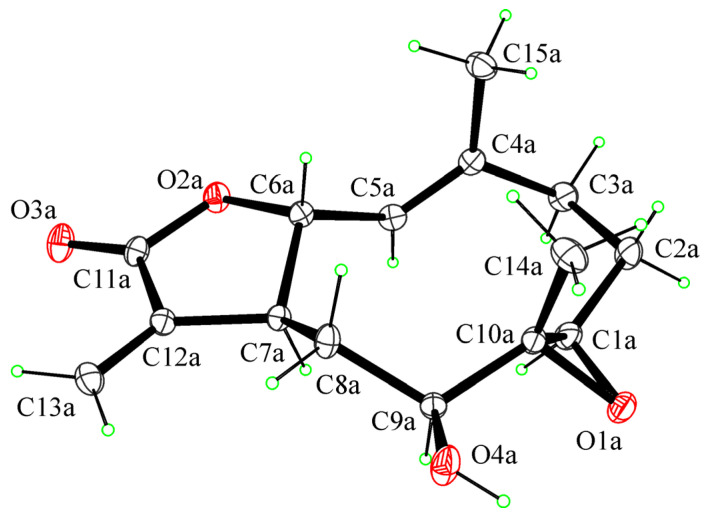
ORTEP view of the molecular structure of **5** with thermal ellipsoids drawn at 30% probability level.

For compound **5**, the relative configuration was confirmed by an X-ray analysis carried out on the single crystal obtained by the slow evaporation of CHCl_3_-*i*-PrOH (1/1, *v*/*v*) in a water-saturated atmosphere at 5 °C. The ORTEP view of the epoxyhaagenolide molecular structure is reported in Figure 3.

The 1*R*, 6*R*, 7*S*, 9*S*, and 10*R* absolute configurations of **5** at the C1, C6, C7, C9, and C10 stereogenic centers were determined on the basis of the anomalous scattering in the X-ray diffraction data by performing X-ray diffraction data collection according to the literature methods reported to assigning the absolute configuration in light-atom structures when MoKα radiation is used (see Section 3 for details).

As shown in Figure 3, the molecule is a sesquiterpene lactone with a 10-membered ring transfused with a 5-membered lactone ring. The lactone ring is in the conformation twisted on C6–C7. The 10-membered ring adopts the chair–chair–chair conformation with methyl groups in axial positions and hydroxy and 1,10-epoxy groups in equatorial positions (see the Supplementary Materials for crystallographic data).

Sesquiterpenes **1**–**5** were independently assayed against *E. faecalis* reference strains and four clinical isolates typed by studying the susceptibility profile to antibiotics (Table 8). 

Compounds were tested by broth microdilution method in a concentration range between 300 and 9 µg/mL. At the highest tested concentrations of 150 and 300 µg/mL, compounds **3**, **4**, and **5** showed activity against all tested strains while compound **2** was active on four of the five tested strains. Growth inhibition rates ranged from 50 to 90% (Table 9). Compound **1**, on the other hand, did not show inhibitory activity against any of the test strains.

Considering the activity in the subgroup of guaianolides (**1**–**3**), the substituent at C-10 seems not important for the activity, while an important structural feature appeared to be the configuration. In fact, the presence of a hydroxy group protruding from the α-side determined inactivity as observed in **1**. In the subgroup of germacrenolides, the nature of the substituents at C-1 and C-10 did not seem to play a role to impart activity.

As described above, enterococci can form biofilms on foreign biomaterials. Bacterial subpopulations within the biofilm characterized by low metabolism have a reduced absorption of antibiotics, especially for the active molecules on the cell wall as beta-lactams and glycopeptides, making the implant-device infections difficult to treat [47,48,49].

Our interest was to investigate the antibiofilm activity of the five natural metabolites on the test strains of *E. faecalis*, using crystal violet as a staining method for total biofilm biomass quantification. Serial dilutions of compounds **1**–**5** were tested in microplates starting from concentrations corresponding to 1/8 or 1/4 of the concentration inhibiting 80% and 50% of planktonic growth, respectively. The compounds, which did not show inhibitory activity against the planktonic form, were tested starting from the concentration of 300 µg/mL (Table 10).

Compound **1** inhibited biofilm formation by all test strains at a concentration of 150 µg/mL. No significant differences were found between biofilm inhibition percentage at 150 and 300 µg/mL (*p* > 0.005). Compounds **2**–**5** exhibited a strain-dependent activity at the maximum concentration tested, showing a biofilm inhibition ranging from 50 to 70% for most of the test strains.

The *E. faecalis* biofilm is highly resistant to the action of ampicillin, vancomycin, and linezolid, despite prolonged times of treatment [50,51]. Some authors have tested the in vitro efficacy of antibiotic combinations against enterococci in biofilms, and they showed how rifampicin-containing antibiotic regimes, rifampicin–tigecycline combinations, and fosfomycin-based combinations were able to reduce the number of bacteria in biofilms formed within 24 h [52,53,54]. However, to date, management strategies in enterococcal PJI are controversial and nonstandardized as well as those in enterococcal endodontic infections, and the disinfection of root canals with commonly used antimicrobial irrigations is very challenging due to cytotoxicity [55,56,57,58].

In light of these considerations, these preliminary data encourage further studies aimed to reach improved antibiofilm activity by combining these natural metabolites with conventional antibiotics to fight PJI as well as with endodontic irrigating agents for more effective intracanal medicaments against this microorganism.

## 3. Materials and Methods

### 3.1. General Experimental Procedures

^1^H and ^13^C nuclear magnetic resonance (NMR) spectra were recorded at 500 and 400 and at 125 and 100 MHz, respectively, in CDCl_3_ by Varian (Palo Alto, CA, USA) and/or Bruker (Karlsruhe, Germany) spectrometers. The same solvent was used as the internal standard. Carbon multiplicities were determined by DEPT spectra [59]. DEPT, COSY-45, HSQC, and HMBC experiments were performed using Bruker microprograms [59]. HR ESIMS and ESIMS were recorded using LC/MS ESIMS-TOF system (Agilent 6230B, HPLC 1260 Infinity) (Milan, Italy). The HPLC separations were performed using a Phenomenex LUNA (C18 (2) 5u 150 × 4.6 mm) (Torrance, CA, USA). Analytical, preparative, and reverse phase thin layer chromatography TLCs were carried out on silica gel (Merck, Kieselgel 60, F_254_, 0.25, 0.5 mm, and RP-18 F_254s_, respectively) plates (Merck, Darmstadt, Germany). The spots were visualized by exposure to UV radiation, or by spraying first with 10% H_2_SO_4_ in MeOH, and then with 5% phosphomolybdic acid in EtOH, followed by heating at 110 °C for 10 min. Column chromatography (CC) was performed using silica gel (Kieselgel 60, 0.063–0.200 mm) (Merck, Darmstadt, Germany). All the solvents were supplied by Sigma-Aldrich (Milan, Italy). The balance model used was Analytical Precisa ES 225SM-DR (Dietikon, Switzerland).

### 3.2. Plant Material

Whole aerial parts of *Cotula cinerea* plants were collected in winter 2019 from the Oued Souf area located in Southern Algeria, which is characterized by dry weather, Saharan climate (33°21′21″ N 6°51′47″ E; mean annual temperature: 21 °C; mean annual precipitation: 75 mm; elevation: 80 m). Plant samples were dried naturally on laboratory benches at room temperature (24 °C) for 1 month. The dried plant materials were powdered by using a blender and then stored away from humidity in bags of conservation. A specimen of the plant was deposited in the collection of the Nationale Supérieure Agronomique, Département de Zoologie Agricole et Forestière, El-Harrach, Alger, Algeria.

### 3.3. Isolation of Plant Metabolites

Plant material (100 g) was extracted (1 × 500 mL) by H_2_O/MeOH (1/1, *v*/*v*) under stirred conditions at room temperature for 24 h; the suspension was centrifuged and the supernatant extracted by *n*-hexane (3 × 300 mL) and successively with CH_2_Cl_2_ (3 × 300 mL) and, after removing methanol under reduced pressure, with EtOAc (3 × 200 mL). The residue (1.04 g) of CH_2_Cl_2_ organic extract, which showed specific antibacterial activity against *E. faecalis*, was purified by CC eluted with CHCl_3_/*i*-PrOH (9/1, *v*/*v*), yielding ten groups of homogeneous fractions (F1–F10). Among them, the residues of fractions F2, F3, and F4 retained antibiotic activity and were further purified using different steps of CC and TLC. F2 was further purified by CC eluted with petroleum ether/acetone (7/3, *v*/*v*), yielding seven groups of homogeneous fractions. Fractions F2.2 was further purified by TLC eluted with CHCl_3_/*i*-PrOH (95/5, *v*/*v*), giving a pure amorphous solid identified as 6-acetoxy-1α-hydroxyguaiantrienolide (**2**, Rf 0.73, 1.70 mg). F2.3 was further purified by TLC eluted with CHCl_3_/*i*-PrOH (95/5, *v*/*v*), giving five homogeneous fractions. The third one gave a pure amorphous solid identified as 6-acetoxy-1β-hydroxyguaiantrienolide (**1**, Rf 0.45, 5.17 mg). The fourth one was further purified by two successive steps of reverse and direct phase TLC eluted with CH_3_CN/H_2_O (1/1, *v*/*v*), and petroleum ether/acetone (8/2, *v*/*v*), yielding a pure compound identified as 6-acetoxy-10β-hydroxyguaiantrienolide (**3**, Rf 0.37, 1.07 mg). F3 was further purified by CC eluted with petroleum ether/acetone (7/3, *v*/*v*), yielding seven groups of homogeneous fractions. Fractions F3.2 was further purified by TLC eluted with CH_2_Cl_2_/*i*-PrOH (95/5, *v*/*v*), giving a pure amorphous solid identified as haagenolide (**4**, Rf 0.41, 41.7 mg). F3.3 was further purified by reverse-phase TLC eluted with CH_3_CN/H_2_O (1/1, *v*/*v*), yielding a pure compound which crystallized by CHCl_3_/*i*-PrOH (1/1, *v*/*v*), identified as 1,10-epoxyhaagenolide (**5**, Rf 0.53, 9.7 mg). This procedure was repeated a few more times using a total of 1.1 kg of plant material.

The characterization and general procedures are described as follows:

6α-acetoxy-1β-hydroxyguaia-4 (15),10(14),11(13)-trien-8α-12-olide (**1**): ^1^H and ^13^C NMR data: see Table 2; ESIMS (+), *m*/*z* 305 [M + H]^+^.

6α-acetoxy-1α-hydroxyguaia-4 (15),10(14),11(13)-trien-8α-12-olide (**2**): ^1^H and ^13^C NMR data: see Table 3; ESIMS (+), *m*/*z* 305 [M + H]^+^.

6α-acetoxy-10β-hydroxyguaia-4 (15),10(14),11(13)-trien-8α-12-olide (**3**): ^1^H and ^13^C NMR data: see Table 4; ESIMS (+), *m*/*z* 305 [M + H]^+^.

Haagenolide (**4**): ${\left[\alpha \right]}_{D}^{25}$ + 40.0 (*c* 0.4, CHCl_3_) (lit. [44]: ${\left[\alpha \right]}_{D}^{24}$ + 38.6 (*c* 1.0, CHCl_3_). ^1^H and ^13^C NMR data: see Table 5; ESIMS (+), *m*/*z* 249 [M + H]^+^.

1,10-Epoxyhaagenolide (**5**): ${\left[\alpha \right]}_{D}^{25}$ + 10.0 (c 0.4, CHCl_3_) (lit. [45]: ${\left[\alpha \right]}_{D}^{24}$ + 7.5 (*c* 0.24, CDCl_3_). ^1^H and ^13^C NMR data: see Table 6; ESIMS (+), *m*/*z* 265 [M + H]^+^.

9-*O*-(*S*)-α-Methoxy-α-trifluoromethyl-α-phenylacetate (MTPA) ester of haagenolide (**6**): (*R*)-(−)-MPTA-Cl (10 μL) was added to **4** (1.1 mg) dissolved in dry pyridine (200 μL). The reaction was stirred at room temperature for 3 days and then was stopped by adding MeOH. Pyridine was removed by a N_2_ stream as azeotrope formed with C_6_H_6_. The purification of the crude residue by preparative TLC on silica gel, eluted with EtOAc-*n*-hexane (4:6, *v*/*v*), gave **6** as a homogeneous solid (0.70 mg, Rf 0.57): ^1^H NMR, see Table 7.

9-*O*-(*R*)-α-Methoxy-α-trifluoromethyl-α-phenylacetate (MTPA) ester of haagenolide (7): (S)-(+)-MPTA-Cl (10 μL) was added to 4 (1.4 mg) dissolved in dry pyridine (200 μL). The reaction was carried out under the same conditions used for preparing 6 from 4. The purification of the crude residue by preparative TLC on silica gel, eluted with EtOAc-*n*-hexane (4:6, *v*/*v*), gave **7** as a homogeneous solid (0.61 mg, Rf 0.57); ^1^H NMR, see Table 7.

### 3.4. Crystallographic Data of 1,10-Epoxyhaagenolide (**5**)

Single crystals of **5**, which were suitable for X-ray structure analysis, were obtained by slow evaporation of CHCl_3_-*i*-PrOH (1/1, *v*/*v*) in a water-saturated atmosphere at 5 °C. One selected crystal was mounted in flowing N_2_ at 173 K on a Bruker-Nonius Kappa CCD diffractometer equipped with an Oxford Cryostream apparatus (graphite monochromated MoKα radiation λ = 0.71073 Å; CCD rotation images, thick slices; φ and ω scans to fill the asymmetric unit). A semiempirical absorption correction (multiscan, SADABS) was applied. The structure was solved by direct methods using SIR97 program [60] and anisotropically refined by the full matrix least-squares method on F^2^ against all independent measured reflections using the SHELXL-2018/3 [61] and WinGX software [62]. Crystallization solvent water molecules were found in the crystal structure. All the hydroxy and water H atoms were individuated in difference Fourier maps and freely refined with *U*_iso_(H) equal to 1.2 U_eq_ of the carrier atom. All the other H atoms were placed in calculated positions and refined accordingly to a riding model with C–H distances in the range 0.95–1.00 Å and U_iso_(H) equal to 1.2 U_eq_ of the carrier atom (1.5 U_eq_ for C_methyl_). The 1*R*, 6*R*, 7*S*, 9*S*, and 10*R* absolute configurations of **5** at C1, C6, C7, C9, and C10 stereogenic centers were determined by performing X-ray diffraction data collection according to the literature methods reported in order to assign the absolute configuration in light-atom structures when MoKα radiation is used. The calculated absolute parameters and the Bayesian statistics *p* values indicate a very high probability that the absolute configuration is correctly assigned [63,64,65]. Bijvoet-pair analysis and Bayesian statistics were performed using the program PLATON [66]. The figures were generated using ORTEP-3 [62] and Mercury CSD 4.0 [67]. Structural details are reported in the Supplementary Materials. The crystallographic data for **5** were deposited in the Cambridge Crystallographic Data Centre with deposition numbers CCDC 2076390. These data can be obtained free of charge from the Cambridge Crystallographic Data Centre via www.ccdc.cam.ac.uk/data_request/cif (accessed on 5 July 2021).

*Crystallographic Data of***5**: (C_15_H_20_O_4_)_2_·H_2_O, M = 546.63, orthorhombic, P2_1_2_1_2_1_, *a* = 7.6570(15) Å, *b* = 14.693(3) Å, *c* = 25.155(3) Å, α = β = γ = 90°, V = 2830.0(9) Å^3^, T = 173(2) K, Z = 4, D_calcd_ = 1.283 Mg/m^3^, crystal size 0.30 × 0.050 × 0.010 mm, F(000) = 1176, absorption coefficient 0.094 mm^−1^, reflections collected 78093, independent reflections 8560 [R_int_ = 0.0502], final R indices [I > 2σ(I)], R1 = 0.0455, wR2 = 0.0897, R indices (all data), R1 = 0.0667, wR2 = 0.1003. Absolute parameters: Flack *x* determined using 2540 quotients: 0.1(2); Parsons *z*: 0.1(2); Bayesian statistics: P2(true) = 1.00, P3(true) = 0.896, P3(rac-twin) = 0.104, P3(false) = 0.7 × 10^−7^, Hooft *y* = 0.16(14), Bijvoet pairs 3724, Friedel coverage 97%. Absolute structure parameters were calculated using the programs SHELXL-2018/3 and PLATON-v30118.

### 3.5. Test Bacterial Strains and Culture Conditions

Bacterial strains used in this study were reference and clinical strains: *Pseudomonas aeruginosa* ATCC 27853, *Acinetobacter baumannii* ATCC 747, *Staphylococcus aureus* ATCC 29213, *Enterococcus faecalis* ATCC 29212, and four clinical strains of *E. faecalis* (EF-91823 and EF-91804 isolated from PJI; EF-165 and EF-91705 isolated from bloodstream infections). The strains were obtained from a collection of anonymous clinical isolates previously established at the Department of Molecular Medicine and Medical Biotechnologies (University of Naples Federico II). No ethical approval was required for the study because there was no access to patients’ data. All strains were stored as 15% (*v*/*v*) glycerol stocks at −80 °C. Before each experiment, cells were subcultured from the stocks onto TSA plates at 37 °C for 24 h. The strains were identified by MS MALDI-TOF (Bruker Daltonics, Bremen, Germany) and were characterized by studying the antibiotic susceptibility profile performed on Vitek 2 (bioMérieux, Marcy-l’Étoile, France).

### 3.6. Antimicrobial Assays

Standard broth microdilution method in 96-well polystyrene plates was used to determine the minimum inhibitory concentration (MIC) of plant extracts and secondary metabolites using Mueller-Hinton Broth 2 (MHB2) as culture medium. For each test strain, starting from bacterial suspensions with a turbidity of 0.5 McFarland (corresponding to 1–5 × 10^8^ cells/mL) and subsequently adjusted to approximately 5 × 10^6^ CFU/mL^−1^, 100 μL aliquots of the suspension were dispensed in wells in triplicate. Then 100 µL of 500 µg/mL of plant extract solution were added to the wells, and plates were incubated at 37 °C for 19 h under shaking (300 rpm) and under aerobic conditions, except for *E. faecalis* strains, which were incubated under anaerobic conditions. Instead, to test the activity of the five metabolites, the wells were added with equal volumes (100 μL) of the suspensions and twofold serial dilutions starting from 300 µg/mL for each metabolite. Wells with no compounds were used as positive controls (100% growth). Conventional antibiotics as ampicillin and amikacin, selected depending on antibiotic-susceptibility profiles of the test strains, were included as control for Gram positive and Gram negative, respectively. The medium turbidity was measured by a spectrophotometer at 595 nm (Bio-Rad Laboratories S.r.l., Hercules, CA, USA). Antimicrobial activity was expressed as a percentage of microbial growth inhibition. Each assay was performed twice. To be sure that the 2% of DMSO present in the 2× stock solutions of the plant extracts and that metabolites did not act on bacterial growth, the effect of serial dilutions of DMSO starting from 1% on the growth of test strains was separately tested.

### 3.7. Biofilm Formation Inhibition Assay

Biofilm biomass formed in vitro in the presence of the five secondary metabolites was measured using the crystal violet (CV) staining method in flat-bottomed 96-well microplates as described by Stepanović et al. with some modifications [68]. For each strain, a cell suspension in BHI supplemented with 1% (*w*/*v*) glucose was prepared and diluted to obtain a suspension of 1.5 × 10^6^ CFU/mL. One hundred microliters of this suspension were incubated with 100 µL of serial dilutions of sub-MIC compounds concentrations, showing no influence on planktonic growth of the test strains. The positive controls were compound-free wells. The microtiter was incubated at 37 °C for 24 h. The nonadherent cells were then removed with gentle aspiration and gentle washings with PBS, and the biofilm was dried at 60 °C for 30 min and subsequently stained with 0.1% (*w*/*v*) crystal violet solution for 30 min. After washing with PBS and solubilization with absolute ethanol to release the dye from the biofilm, absorbance measurement values at 570 nm were obtained using the spectrophotometric reading. The absorbance recorded was correlated to the amount of biofilm produced. The percentage of biofilm mass reduction was calculated using the formula [(Ac-At)/Ac] × 100, where Ac is the OD570 for control wells, and At is the OD570 in the presence of the tested compound.

### 3.8. Statistical Analysis

Data were represented as the mean ± standard deviation and analyzed for statistical significance using ordinary one-way analysis of variance (ANOVA) and Tukey’s multiple comparisons test. For all tests, *p* < 0.005 was considered to indicate a statistically significant difference.

## 4. Conclusions

This is the first report on the antimicrobial and antibiofilm activities of three guaiantrienolides and two germacrenolides isolated from *Cotula cinerea* against clinical isolates of *Enterococcus faecalis*, a common drug-resistant opportunistic pathogen responsible for important biofilm-related infections. Our results are preliminary data, but we can hypothesize that these natural substances could be a potential alternative for new antibiofilm formulations in strategies for the prevention of persistent infection by *Enterococcus faecalis*. The introduction of modifications in their structure on the basis of structure–function studies could lead to the synthesis of derivatives with higher antibiofilm activity. In addition, the absolute configuration of two of the three germacranolides was also assigned.

## Figures and Tables

**Table 1:** Antibacterial activity of CH_2_Cl_2_ extract (500 µg/mL), expressed as percentage of growth inhibition, with ± standard deviation (SD), against Gram-positive and Gram-negative test strains (n.d. = inhibition not detected).

Strain	CH_2_Cl_2_ Extract % Inhibition
*S. aureus* ATCC 29213	49 ± 2.7
*E. faecalis* ATCC 29212	90 ± 1.5
*P. aeruginosa* ATCC 27853	n.d.
*A. baumannii* ATCC 747	n.d.

**Table 2:** ^1^H and ^13^C NMR data of 6α-acetoxy-1β-hydroxyguaia-4 (15),10(14),11(13)-trien-8α-12-olide (**1**) *^a,b^*.

Position	δC *^c^*	δH (*J* in Hz)	HMBC
1	84.6 s		H_2_-14, H-9B, H-6, H-2B
2	37.4 t	2.32 ddd (12.0, 8.7, 3.5) 1.88 br dd (12.0, 7.8, 1.5)	
3	29.8 t	2.68 m 2.43 br dd (15.8, 8.7)	H_2_-15, H-2A
4	149.1 s		H-6, H-2B
5	55.4 d	2.78 m	H_2_-15, H-6
6	73.2 d	5.49 dd (5.9, 4.1)	H-8, H-7
7	48.5 d	3.09 br dd (10.9, 5.9)	H_2_-13, H-9A, H-6
8	75.6 d	4.62 dt (10.9, 8.0)	H_2_-9
9	38.3 t	3.58 ddt (14.2, 8.0, 1.7) 2.51 dd (14.2, 8.0)	
10	145.6		H_2_-9
11	169.5		H_2_-13
12	137.5		H-13A, H-6
13	122.2	6.28 d (4.4) 5.95 d (4.4)	
14	116.9	5.37 br s 5.15 br s	H_2_-9
15	109.3	5.14 br s 4.98 br s	
CH_3_CO	169.5		CH_3_CO, H-6
CH_3_CO	20.9	2.02 s	

*^a^* The chemical shifts are in δ values (ppm) from TMS. *^b^* 2D ^1^H, ^1^H (COSY) ^13^C, and ^1^H (HSQC) NMR experiments delineated the correlations of all the protons and the corresponding carbons. *^c^* Multiplicities were assigned by DEPT spectrum.

**Table 3:** ^1^H and ^13^C NMR data of 6α-acetoxy-1α-hydroxyguaia-4 (15),10(14),11(13)-trien-8α-12-olide (**2**) *^a,b^*.

Position	δC ^*c*^	δH (*J* in Hz)	HMBC
1	84.3 s		H_2_-14, H-6, H-3A, H-2B
2	37.1 t	2.16 br dd (12.7, 6.8) 1.84 br dd (12.7, 6.3)	
3	31.9 t	2.47 br d (15.0, 6.8) 1.34 m	
4	147.9 s		
5	54.6 d	2.68	H-3A, H-2B
6	72.3 d	5.87 dd (7.8, 5.2)	
7	51.6 d	3.73 br dd (10.0, 7.8)	H_2_-13, H_2_-9
8	77.8 d	4.18 td (10.0, 3.8)	H_2_-9
9	41.3 t	3.06 (2H) m	H_2_-14
10	146.0 s		H_2_-9
11	169.1 s		H_2_-13
12	138.1 s		H-13A, H-6
13	121.6 t	6.25 d (4.1) 5.76 d (4.1)	
14	113.6 t	5.17 br s 5.08 br s	H_2_-9
15	109.0 t	5.14 br s 4.85 br s	
CH_3_CO	170.0 s		CH_3_CO, H-6
CH_3_CO	21.2 q	2.10	

*^a^* The chemical shifts are in δ values (ppm) from TMS. *^b^* 2D ^1^H, ^1^H (COSY) ^13^C, and ^1^H (HSQC) NMR experiments delineated the correlations of all the protons and the corresponding carbons. *^c^* Multiplicities were assigned by DEPT spectrum.

**Table 4:** ^1^H and ^13^C NMR data of 6α-acetoxy-10β-hydroxyguaia-4 (15),10(14),11(13)-trien-8α-12-olide (**3**) *^a,b^*.

Position	δC *^c^*	δH (J in Hz)	HMBC
1	146.9 s		Me-14, H-9, H-6, H-5
2	137.7 d	6.04 br s	H-5
3	39.1 t	3.15 2.88 m ^d^	H_2_-15
4	148.7 s		H-5
5	50.7 d	3.80 br d (6.8)	H_2_-15
6	75.0 d	5.65 dd (7.8, 6.8)	
7	51.4 d	2.92 m^d^	H-9A
8	74.7 d	4.59 br t (10.5, 2.3)	H_2_-9
9	48.4 t	2.61 dd (13.2, 2.3) 1.80 br t (13.0)	Me-14
10	69.4 s		Me-14, H-9
11	169.4 s		H_2_-13
12	137.7 s		H_2_-13
13	122.2 t	6.25 d (3.3) 5.69 d (3.3)	
14	29.4 q	1.61 br s	
15	109.8 t	5.05 br s 4.96 br s	H-5
CH_3_CO	170.1 s		CH_3_CO, H-6
CH_3_CO	21.3 q	2.07	

*^a^* The chemical shifts are in δ values (ppm) from TMS. *^b^* 2D ^1^H, ^1^H (COSY) ^13^C, and ^1^H (HSQC) NMR experiments delineated the correlations of all the protons and the corresponding carbons. *^c^* Multiplicities were assigned by DEPT spectrum.

**Table 5:** ^1^H and ^13^C NMR data of haagenolide (**4**) *^a,b^*.

Position	δC ^*c*^	δH (*J* in Hz)	HMBC
1	129.4 d	5.12 br d (10.7, 1.2)	H_2_-3, H-9, Me-14
2	35.6 t	2.00 m	H_2_-3, Me-15
3	39.4 t	2.38 br t (11.7) 2.07 td (11.7, 4.6)	H-5, Me-15
4	141.3 s		H-6, Me-15
5	127.1 d	4.65 d (9.9)	H-3A, H-6, Me-15
6	81.3 d	4.59 dd (9.9)	H_2_-8
7	47.3 d	2.69 br td (9.9, 3,5)	H-5, H_2_-8, H_2_-13,
8	25.5 t	2.31 br t (13.8) 1.95 ddd (13.8, 10.6, 2.2)	H-6
9	79.7 d	4.26 dd (10.6, 2.2)	H-1, H_2_-8, Me-14
10	139.0 s	-	H-2, H_2_-8 Me-14
11	170.0 s	-	H_2_-13
12	139.2 s	-	H_2_-13
13	120.1 t	6.31 d (3.5) 5.61 d (3.5)	H-7
14	10.9 q	1.48 s	H-1, H-9
15	17.5 q	1.75 s	H_2_-3, H-5

*^a^* The chemical shifts are in δ values (ppm) from TMS. *^b^* 2D ^1^H, ^1^H (COSY) ^13^C, and ^1^H (HSQC) NMR experiments delineated the correlations of all the protons and the corresponding carbons. *^c^* Multiplicities were assigned by DEPT spectrum.

**Table 6:** ^1^H and ^13^C NMR data of 1,10-epoxyhaageanolide (**5**) *^a,b^*.

Position	δC ^*c*^	δH (*J* in Hz)	HMBC
1	67.1 d	2.90 dd (11.2, 2.3)	H-9, H-3B
2	23.4 t	2.14 br ddd (13.7, 4.8, 2.3) 1.50 br ddd (13.7, 11.2, 4.8)	H-3A, H-1
3	35.9 t	2.43 d t (12.5, 4.8) 2.21 m	H-5, Me-15
4	144.6 s		H-6, H-3B, Me-15
5	123.9 d	5.21 d (10.2)	H-3A, Me-15
6	80.2 d	4.62 t (10.2)	H_2_-8
7	47.2 d	2.70 br t (9.5)	
8	32.9 t	2.29 br d (15.0, 2.0) 1.79 dt (15.0, 9.5)	H-5, H-6, H2-8, H2-13
9	79.9 d	3.24 dd (9.5, 2.0)	H_2_-8, Me-14
10	64.7 s		H_2_-8 Me-14
11	170.0 s		H_2_-13
12	138.7 s		H-13A
13	120.1 t	6.31 d (3.5) 5.61 d (3.5)	
14	11.8 q	1.48 s	H-9
15	17.9 q	1.75 s	H-3A

*^a^* The chemical shifts are in δ values (ppm) from TMS. *^b^* 2D ^1^H, ^1^H (COSY) ^13^C, and ^1^H (HSQC) NMR experiments delineated the correlations of all the protons and the corresponding carbons. *^c^* Multiplicities were assigned by DEPT spectrum.

**Table 7:** ^1^H NMR data of *R*-MTPA (**6**) and *S*-MTPA (**7**) monoesters *^a^*.

	6	7
Position	δH	δH
2	2.310 (2H)	2.296 (2H)
3	2.388 1.982	2.379 2.058
5	4.678	4.668
6	4.551	4.562
7	2.753	2.757
8	2.090 2.019	2.198 2.100
9	5.482	5.454
13	6.299 5.482	6.313 5.510
14	1.436	1.304
15	1.725	1.713
OMe	3.519	3.557
Ph	7.508–7.384	7.532–7.367

*^a^* The chemical shifts are in δ values (ppm) from TMS.

**Table 8:** Antibiotic susceptibility profile performed to Vitek 2 system (bioMérieux) of four clinical isolates of *E. faecalis* and reference strain *E. faecalis* ATCC29212.

Strains	Minimum Inhibitory Concentration (µg/mL)					
	AMP	IM	LIN	TEI	VAN	TIG
EF-91823	≤2	≤2	2	≤0.5	2	≤0.25
EF-91804	≤2	≤2	≤0.5	≤0.5	1	≤0.25
EF-165	≤2	≤1	2	≤0.5	2	≤0.12
EF-91705	≤2	≤1	2	≤0.5	1	0.25
ATTC29212	≤2	≤1	2	≤0.5	2	≤0.12

AMP = ampicillin, IM = imipenem, LIN = linezolid, TEI = teicoplanin, VAN = vancomycin, TIG = tigecycline.

**Table 9:** Antibacterial activity, expressed as percentage of maximum growth inhibition (GI) (mean percentage ± SD) of compounds **1**–**5** at concentrations ranged from 300 to 9 μg/mL against *E. faecalis* test strains.

Strains	Compound 1		Compound 2		Compound 3		Compound 4		Compound 5	
	µg/mL	%GI	µg/mL	%GI	µg/mL	%GI	µg/mL	%GI	µg/mL	%GI
EF-91804	300	nd	150	78 ± 1.5	150	80 ± 1.1	300	77 ± 1.5	300	82 ± 0.9
EF-91823	300	nd	300	50 ± 1.9	300	51 ± 0.9	300	49 ± 1.1	300	50 ± 1.6
EF-165	300	nd	300	nd	300	90 ± 0.7	300	80 ± 3	300	81 ± 3.3
EF-91705	300	nd	300	49 ± 2.2	300	50 ± 2.3	300	55 ± 2.6	150	80 ± 0.8
ATCC29212	300	nd	300	80 ± 1.1	300	79 ± 1.8	300	70 ± 2.1	300	79 ± 1.5

**Table 10:** In vitro biofilm formation inhibition (BI) of *E. faecalis* test strains, presented as mean percentage ± SD, following overnight treatment with compounds **1**–**5** at serial dilutions of sub-planktonic growth inhibitory concentrations.

Strains	Compound 1		Compound 2		Compound 3		Compound 4		Compound 5	
	µg/mL	%BI	µg/mL	%BI	µg/mL	%BI	µg/mL	%BI	µg/mL	%BI
EF-91804	150	60 ± 2.8	18	n.d.	18	52 ± 2.4	37	n.d.	37	53 ± 2
EF-91823	150	50 ± 2.1	75	49 ± 3.1	75	55 ± 1.9	75	52 ± 3.1	75	73 ± 1.1
EF-165	150	52 ± 1.7	150	50 ± 2.4	37	62 ± 1.1	37	65 ± 1.8	37	50 ± 1.2
EF-91705	150	55 ± 3.3	75	50 ± 3.6	75	48 ± 2.2	75	50 ± 2.2	37	56 ± 3.4
ATCC29212	150	61 ± 4.1	18	n.d.	18	n.d.	37	n.d.	37	n.d.

Biofilm formation was determined by crystal violet assay; n.d. = not detected (no difference detected with respect to the control, *p* > 0.005).

## Data Availability

Data are contained within the text and the Supplementary Materials.

## Supplementary Materials

The following are available online at https://www.mdpi.com/article/10.3390/antibiotics10070819/s1. 1D and 2D ^1^H and ^13^C NMR spectra of compounds **1**–**5** and X-ray data of compound **5**.
