# Supplementary material for: Sesquiterpene Lactones from Cotula cinerea with Antibiotic Activity against Clinical Isolates of Enterococcus faecalis
Source: Antibiotics (Basel). 2021 Jul 6;10(7):819. doi: 10.3390/antibiotics10070819 (PMC8300741; doi:10.3390/antibiotics10070819)
Supplement: Supplementary file 1 [file antibiotics-10-00819-s001.zip › antibiotics-1275773-supplementary.pdf]

Supplementary Materials

Supporting Information List

Page 3: **Figure S1**. ^{1}H NMR spectrum of 6-acetoxy-1β-hydroxyguaiantrienolide, **1** ($CDCl_3$, 400 MHz).

Page 3: **Figure S2**. ^{13}C NMR spectrum of 6-acetoxy-1β-hydroxyguaiantrienolide, **1** ($CDCl_3$, 100 MHz).

Page 4: **Figure S3**. COSY spectrum of 6-acetoxy-1β-hydroxyguaiantrienolide, **1** ($CDCl_3$, 400 MHz).

Page 4: **Figure S4**. HSQC spectrum of 6-acetoxy-1β-hydroxyguaiantrienolide, **1** ($CDCl_3$, 400/100 MHz).

Page 5: **Figure S5**. HMBC spectrum of 6-acetoxy-1β-hydroxyguaiantrienolide, **1** ($CDCl_3$, 400/100 MHz).

Page 5: **Figure S6.** NOESY spectrum of 6-acetoxy-1β-hydroxyguaiantrienolide, **1** ($CDCl_3$, 400 MHz).

Page 6: **Figure S7**. ^{1}H NMR 6-acetoxy-1α-hydroxyguaiantrienolide**,** **2** ($CDCl_3$, 400 MHz).

Page 6: **Figure S8**. ^{13}C NMR spectrum of 6-acetoxy-1α-hydroxyguaiantrienolide, **2** ($CDCl_3$, 100 MHz).

Page 7: **Figure S9**. COSY spectrum of 6-acetoxy-1α-hydroxyguaiantrienolide, **2** ($CDCl_3$, 400 MHz).

Page 7: **Figure S10**. HSQC spectrum of 6-acetoxy-1α-hydroxyguaiantrienolide, **2** ($CDCl_3$, 400/100 MHz).

Page 8: **Figure S11**. HMBC spectrum of 6-acetoxy-1α-hydroxyguaiantrienolide, **2** ($CDCl_3$, 400/100 MHz).

Page 8: **Figure S12.** NOESY spectrum of 6-acetoxy-1α-hydroxyguaiantrienolide, **2** ($CDCl_3$, 400 MHz).

Page 9: **Figure S13**. ^{1}H NMR spectrum of 6-acetoxy-10–β–hydroxyguaiantrienolide, **3** ($CDCl_3$, 400 MHz).

Page 9: **Figure S14**. ^{13}C NMR spectrum of 6-acetoxy-10–β–hydroxyguaiantrienolide, **3** ($CDCl_3$, 100 MHz).

Page 10: **Figure S15**. COSY spectrum of 6-acetoxy-10–β–hydroxyguaiantrienolide, **3** ($CDCl_3$, 400 MHz).

Page 10: **Figure S16**. HSQC spectrum of 6-acetoxy-10–β–hydroxyguaiantrienolide, **3** ($CDCl_3$, 400/100 MHz).

Page 11: **Figure S17**. HMBC spectrum of 6-acetoxy-10–β–hydroxyguaiantrienolide, **3** ($CDCl_3$, 400/100 MHz).

Page 11: **Figure S18.** NOESY spectrum of 6-acetoxy-10–β–hydroxyguaiantrienolide, **3** ($CDCl_3$, 400 MHz).

Page 12: **Figure S19**: ^{1}H NMR spectrum of haagenolide **4**, ($CDCl_3$, 400 MHz).

Page 12: **Figure S20.** ^{13}C NMR spectrum of haagenolide **4**, (CDCl3, 100 MHz).

Page 13: **Figure S21.** COSY spectrum of haagenolide **4**, $CDCl_3$, 400 MHz).

Page 13: **Figure S22.** HSQC spectrum of haagenolide **4**, ($CDCl_3$, 400/100 MHz).

Page 14: **Figure S23.** HMBC spectrum of haagenolide **4**, ($CDCl_3$, 400/100 MHz).

Page 14: **Figure S24**. NOESY spectrum of haagenolide **4**, ($CDCl_3$, 400 MHz).

Page 15: **Figure S25**: ^{1}H NMR spectrum of 1,10-epoxyhaagenolide **5**, ($CDCl_3$, 400 MHz).

Page 15: **Figure S26.** ^{13}C NMR spectrum 1,10-epoxyhaagenolide **5** (CDCl3, 100 MHz).

Page 16: **Figure S27.** COSY spectrum of 1,10-epoxyhaagenolide **5 (**$CDCl_3$, 400 MHz).

Page 16: **Figure S28.** HSQC spectrum of 1,10-epoxyhaagenolide **5** ($CDCl_3$, 400/100 MHz).

Page 17: **Figure S29**. HMBC spectrum of 1,10-epoxyhaagenolide **5** ($CDCl_3$, 400/100 MHz).

Page 17: **Figure S30**. NOESY spectrum of 1,10-epoxyhaagenolide **5** ($CDCl_3$, 400 MHz).

Page 18: **Figure S31.** Perspective view of the crystallographic independent unit of **5.**

Page 18: **Figure S32**. Perspective view of **5** with superimposition of the two independent molecules A (element colours) and B (orange).

Page 19: **Figure S33.** Crystal packing of **5** viewed down **a** axis.

Page 19: **Table S1.** Crystal data and structure refinement parameters for **5**.

Page 20: **Table S2.** Selected bond lengths [Å] for (**5**).

Page 20: **Table S3.** Hydrogen bonds for **5** [Å and °].

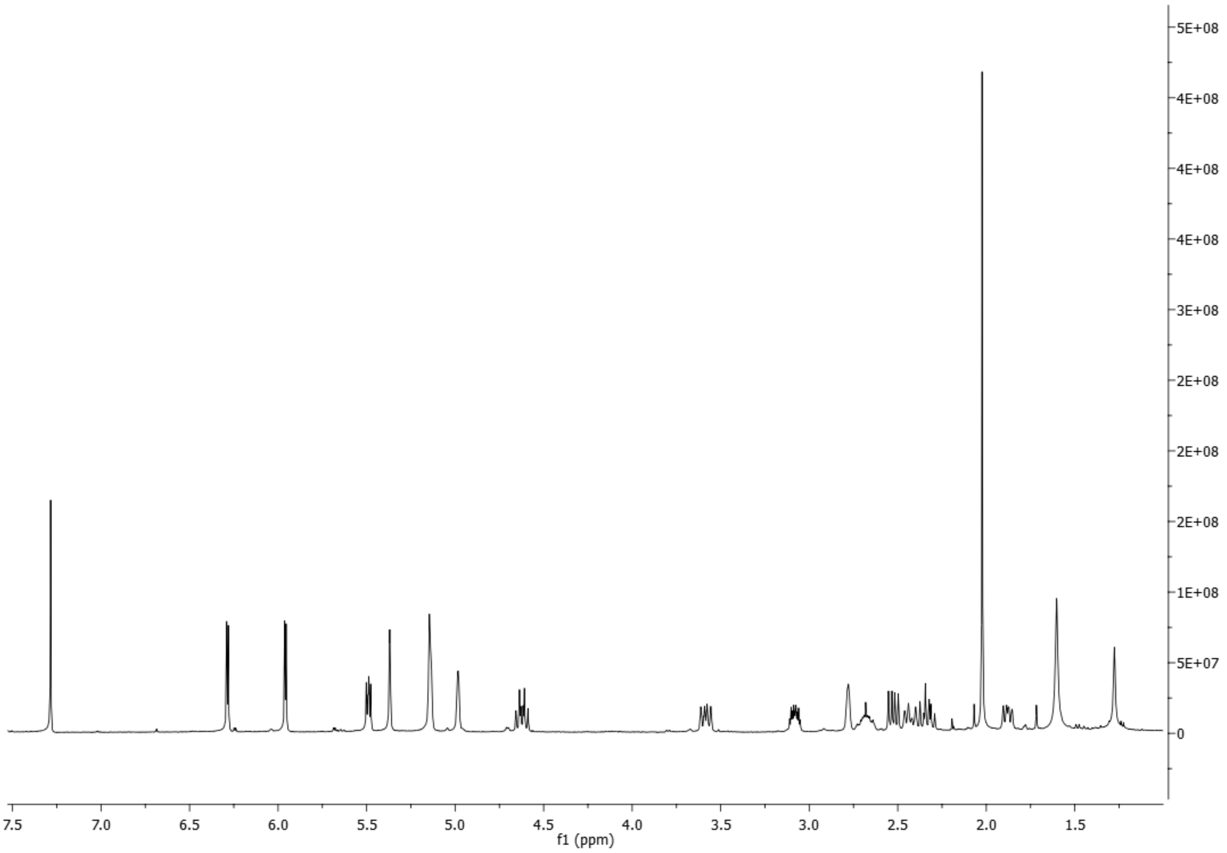

Figure S1. ^{1}H NMR spectrum of 6-acetoxy-1β-hydroxyguaiantrienolide, **1** ($CDCl_3$, 400 MHz).

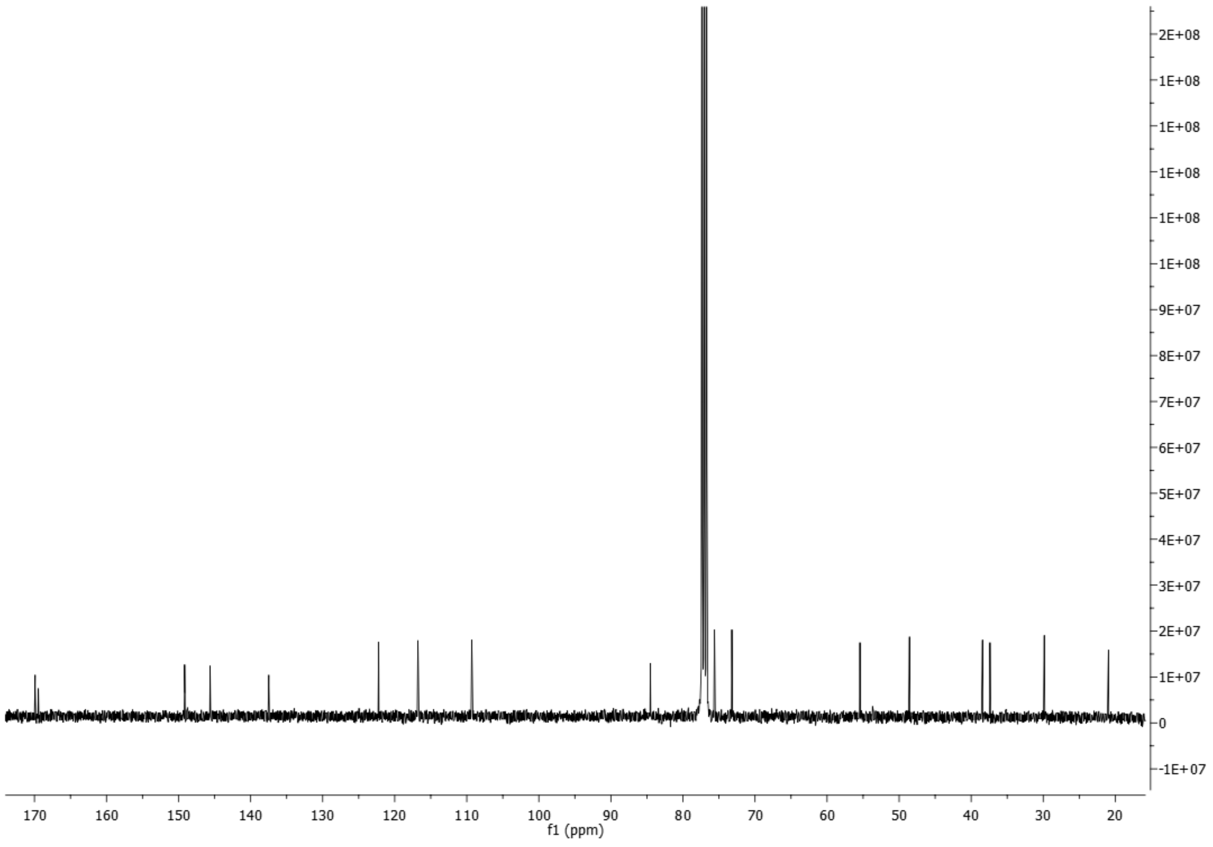

Figure S2. ^{13}C NMR spectrum of 6-acetoxy-1β-hydroxyguaiantrienolide, **1** ($CDCl_3$, 100 MHz).

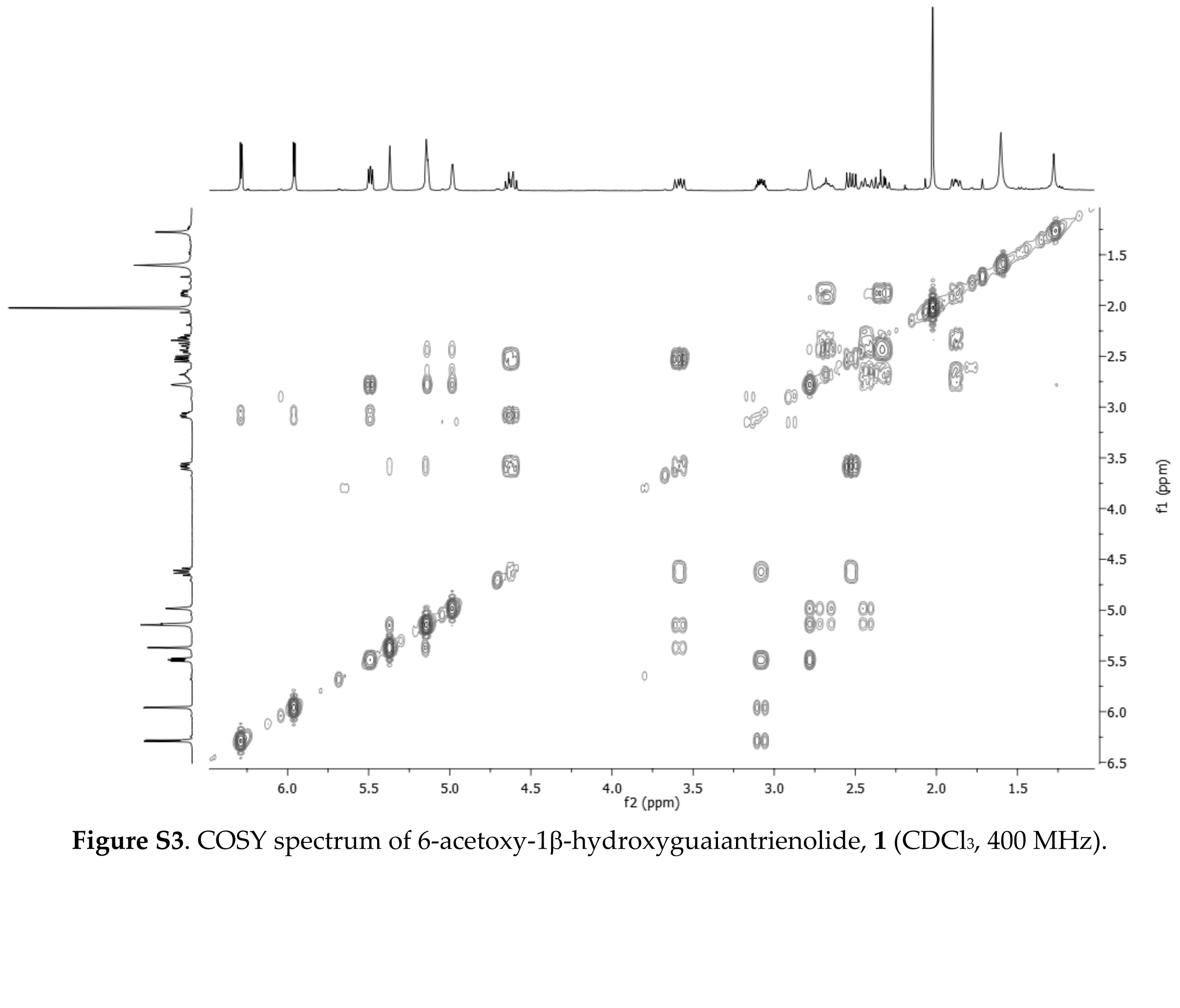

Figure S3. COSY spectrum of 6-acetoxy-1β-hydroxyguaiantrienolide, **1** ($CDCl_3$, 400 MHz).

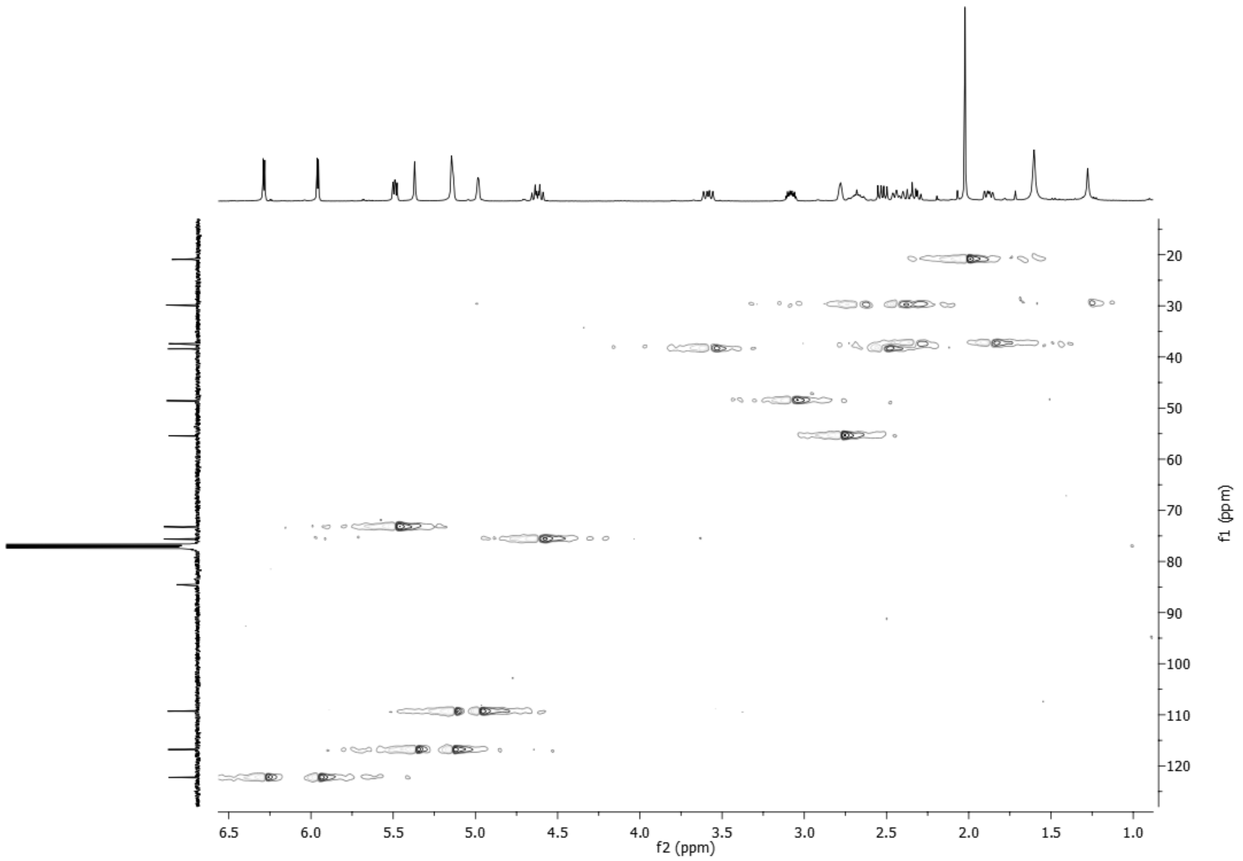

Figure S4. HSQC spectrum of 6-acetoxy-1β-hydroxyguaiantrienolide, **1** ($CDCl_3$, 400/100 MHz)

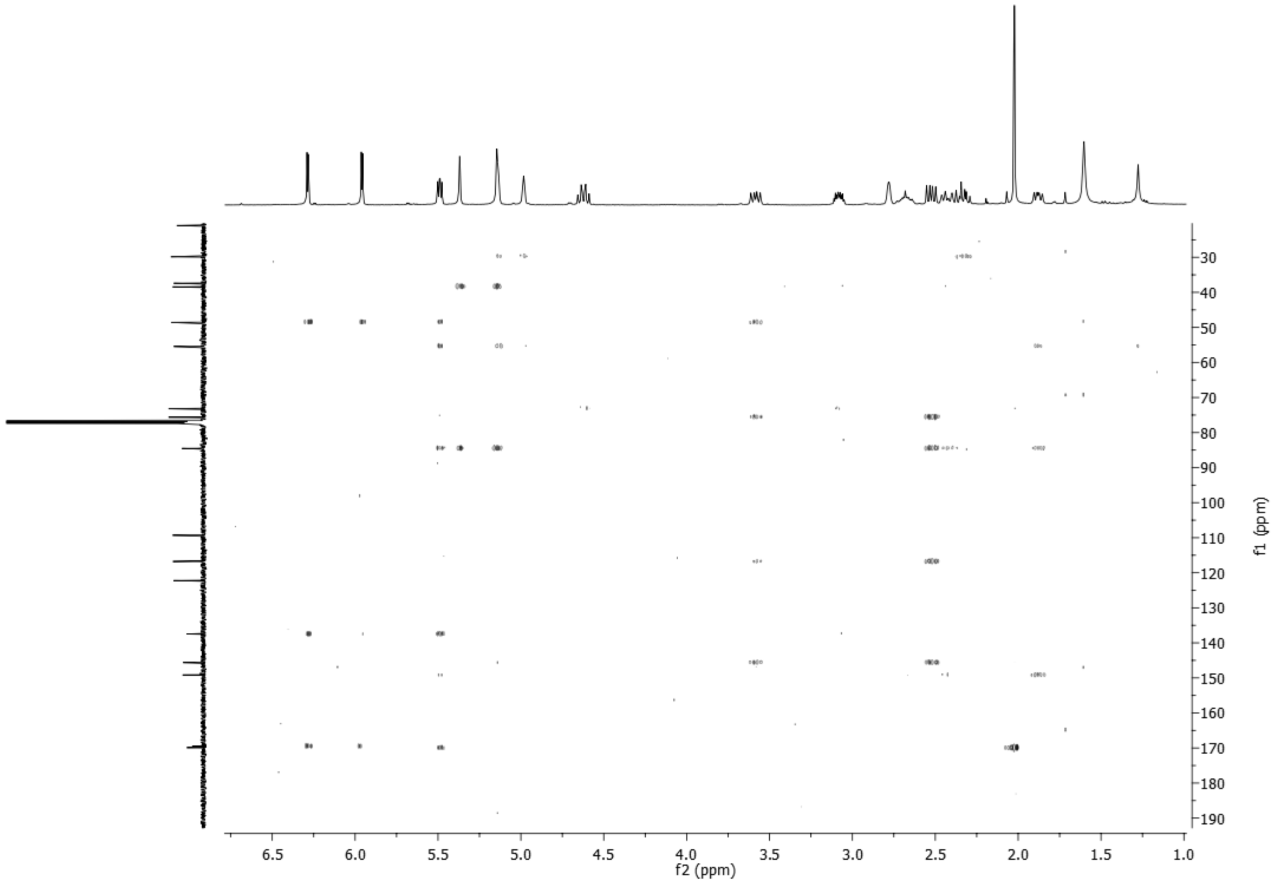

Figure S5. HMBC spectrum of 6-acetoxy-1β-hydroxyguaiantrienolide, **1** ($CDCl_3$, 400/100 MHz).

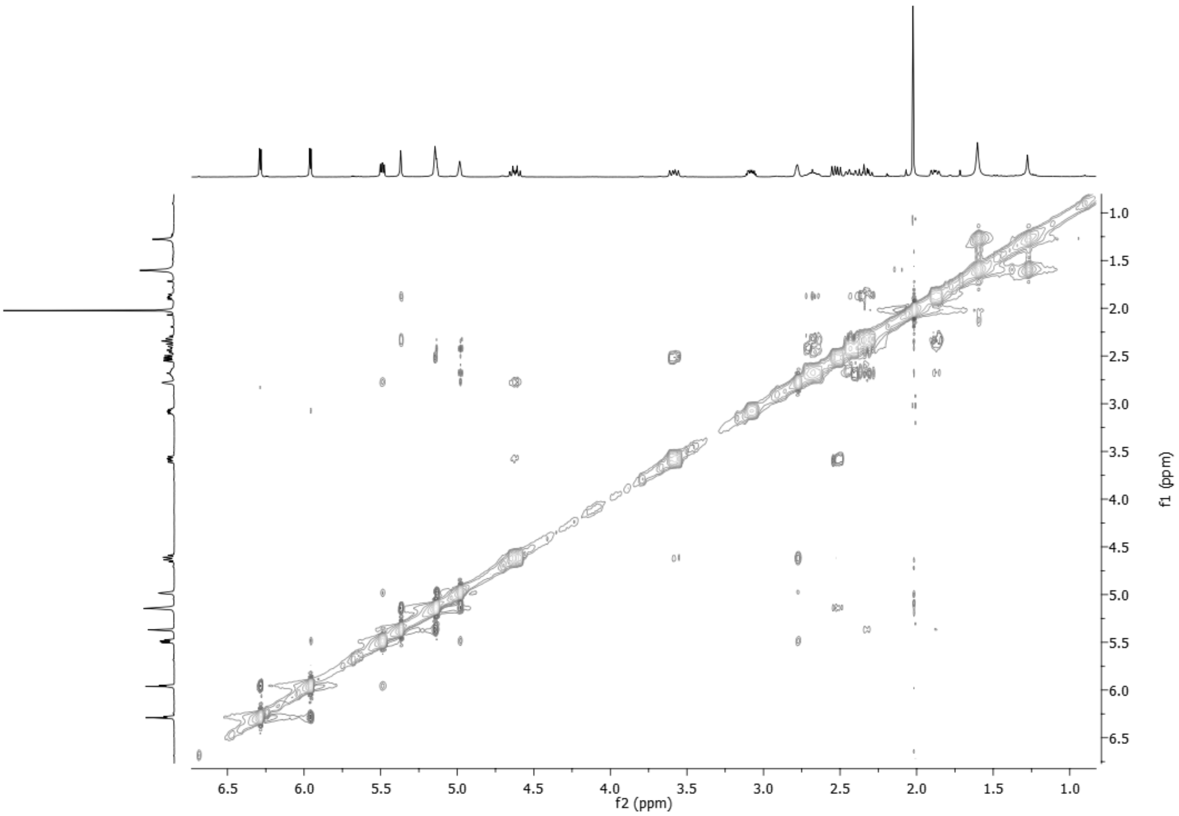

Figure S6. NOESY spectrum of 6-acetoxy-1β-hydroxyguaiantrienolide, **1** ($CDCl_3$, 400 MHz).

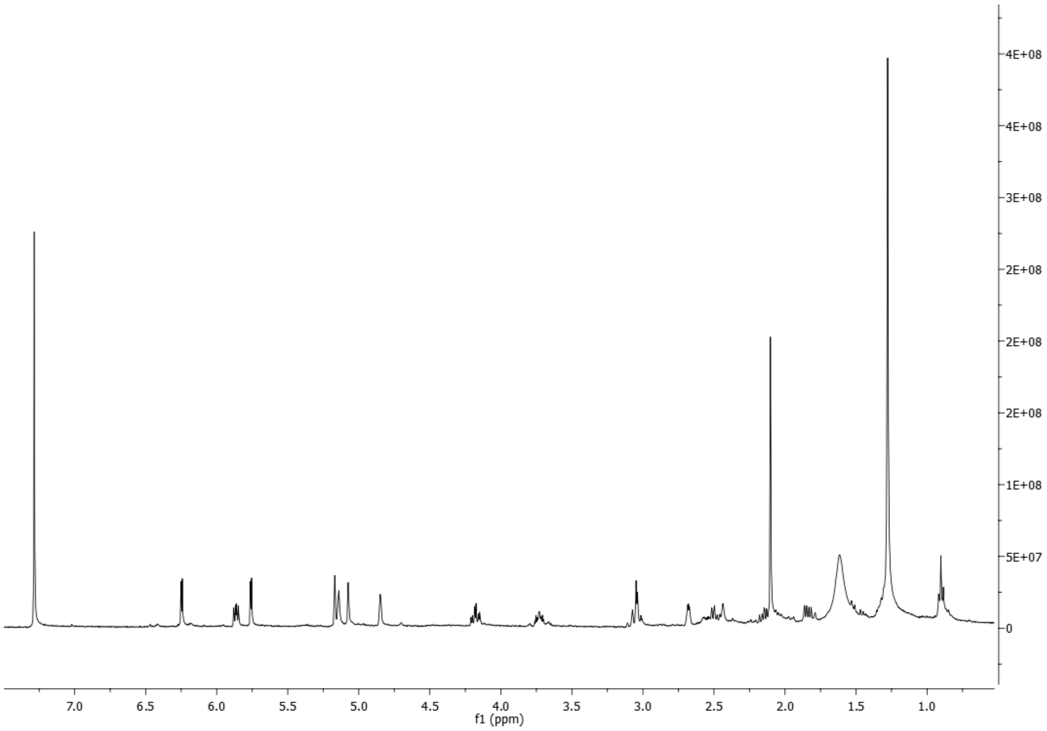

Figure S7. ^{1}H NMR spectrum of 6-acetoxy-1α-hydroxyguaiantrienolide, **2** ($CDCl_3$, 400 MHz).

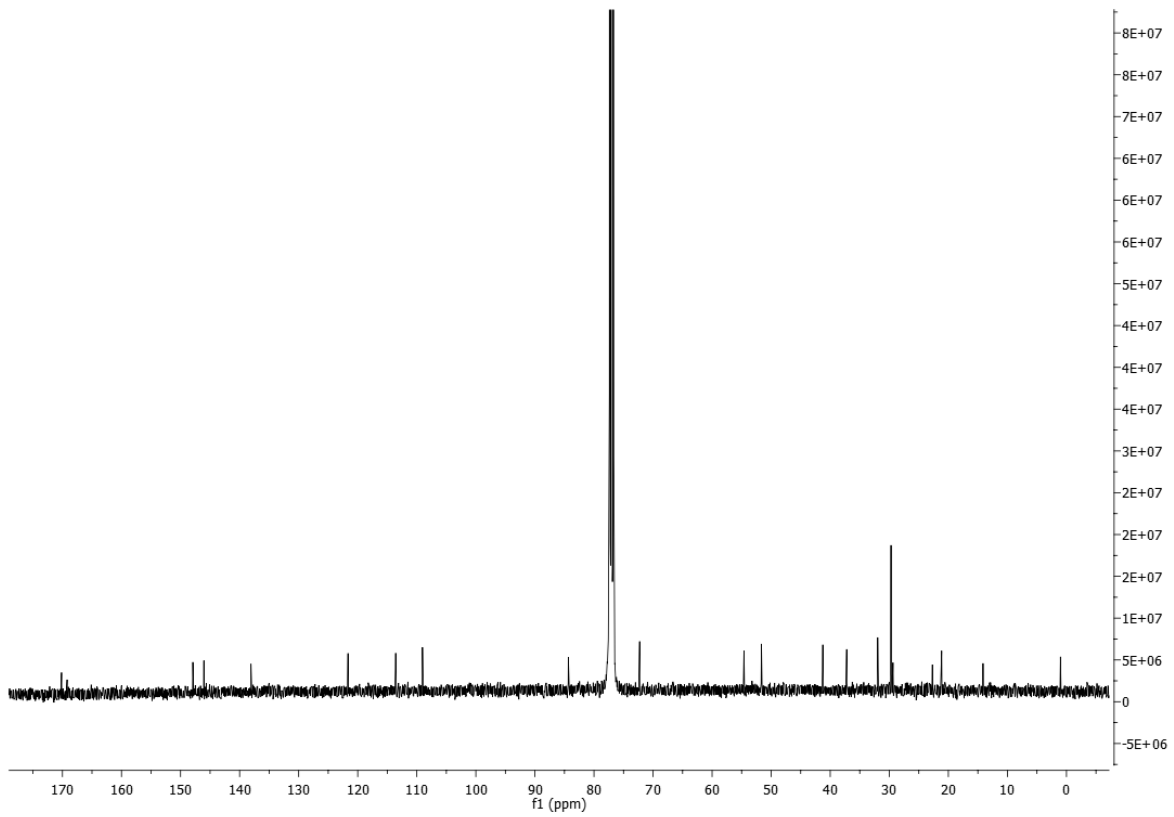

Figure S8. ^{13}C NMR spectrum of 6-acetoxy-1α-hydroxyguaiantrienolide

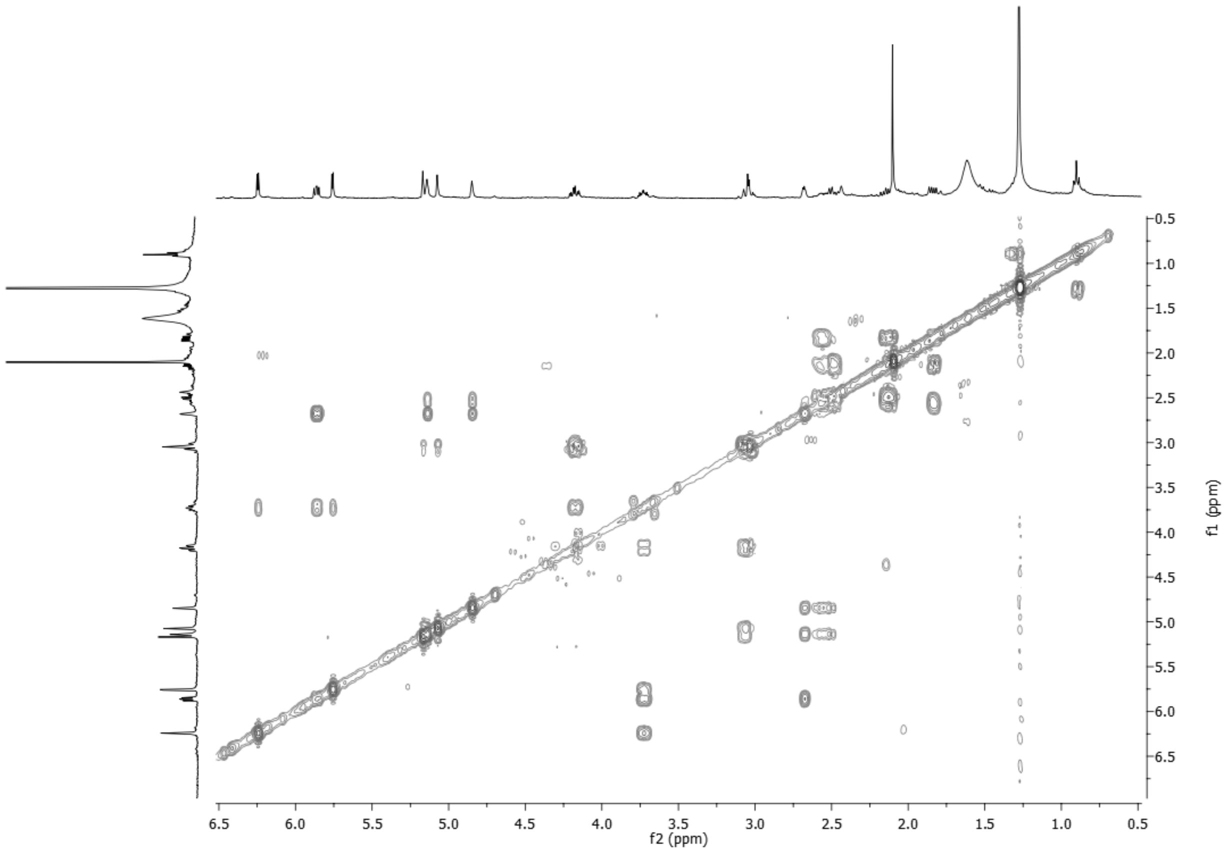

Figure S9. COSY spectrum of 6-acetoxy-1α-hydroxyguaiantrienolide, **2** ($CDCl_3$, 400 MHz).

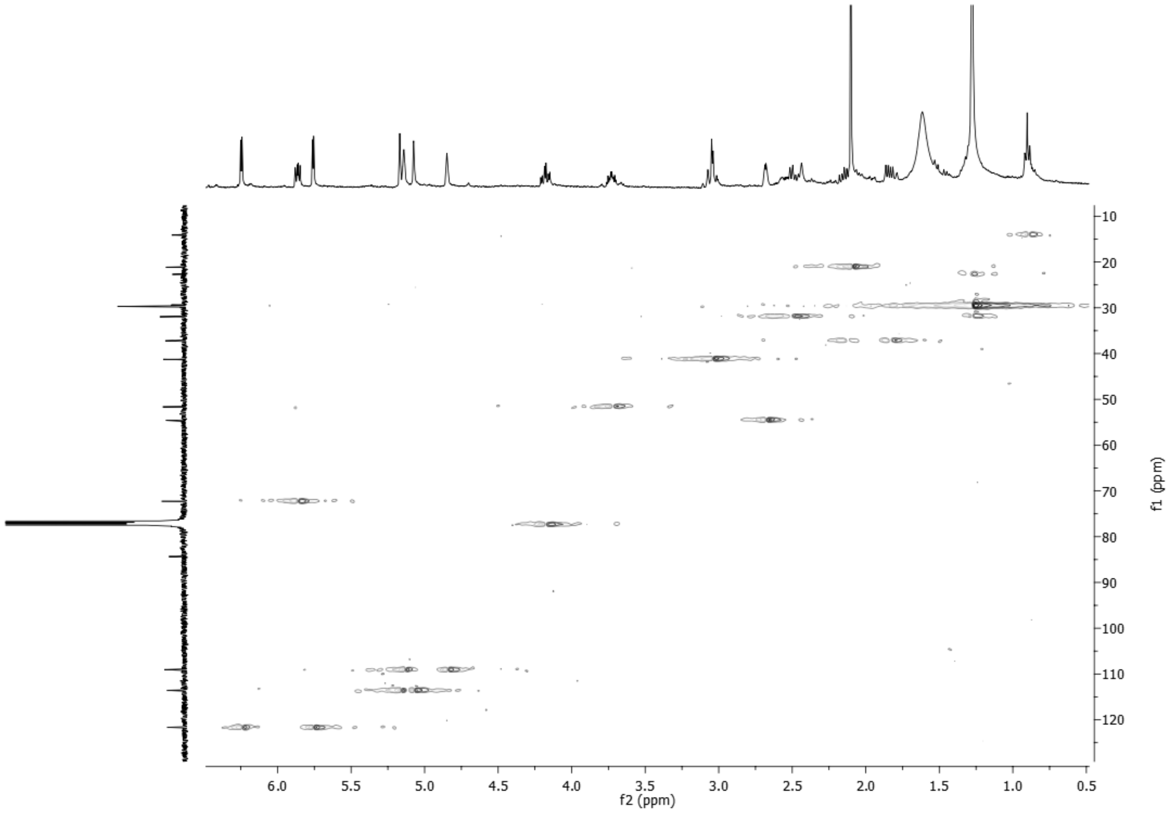

Figure S10. HSQC spectrum of 6-acetoxy-1α-hydroxyguaiantrienolide, **2** ($CDCl_3$, 400/100 MHz).

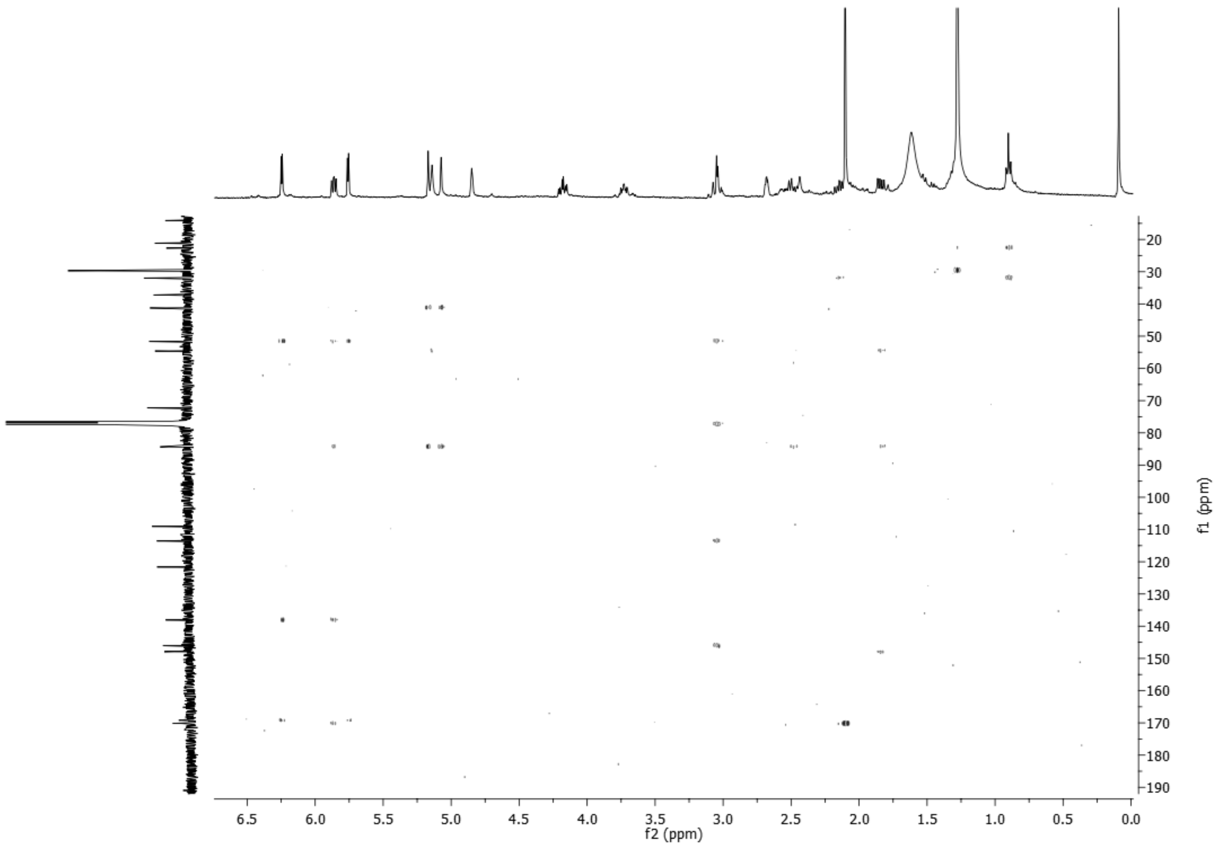

Figure S11. HMBC spectrum of 6-acetoxy-1α-hydroxyguaiantrienolide, **2** ($CDCl_3$, 400/100 MHz).

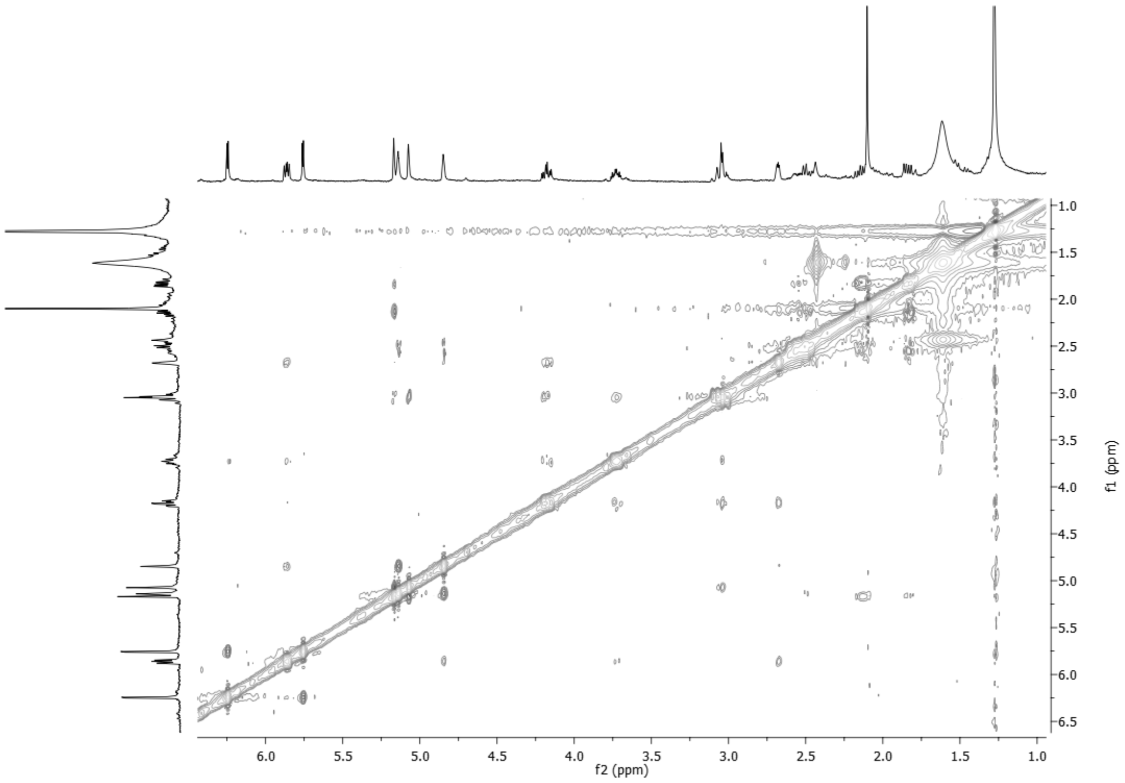

Figure S12. NOESY spectrum of 6-acetoxy-1α-hydroxyguaiantrienolide, **2** ($CDCl_3$, 400 MHz).

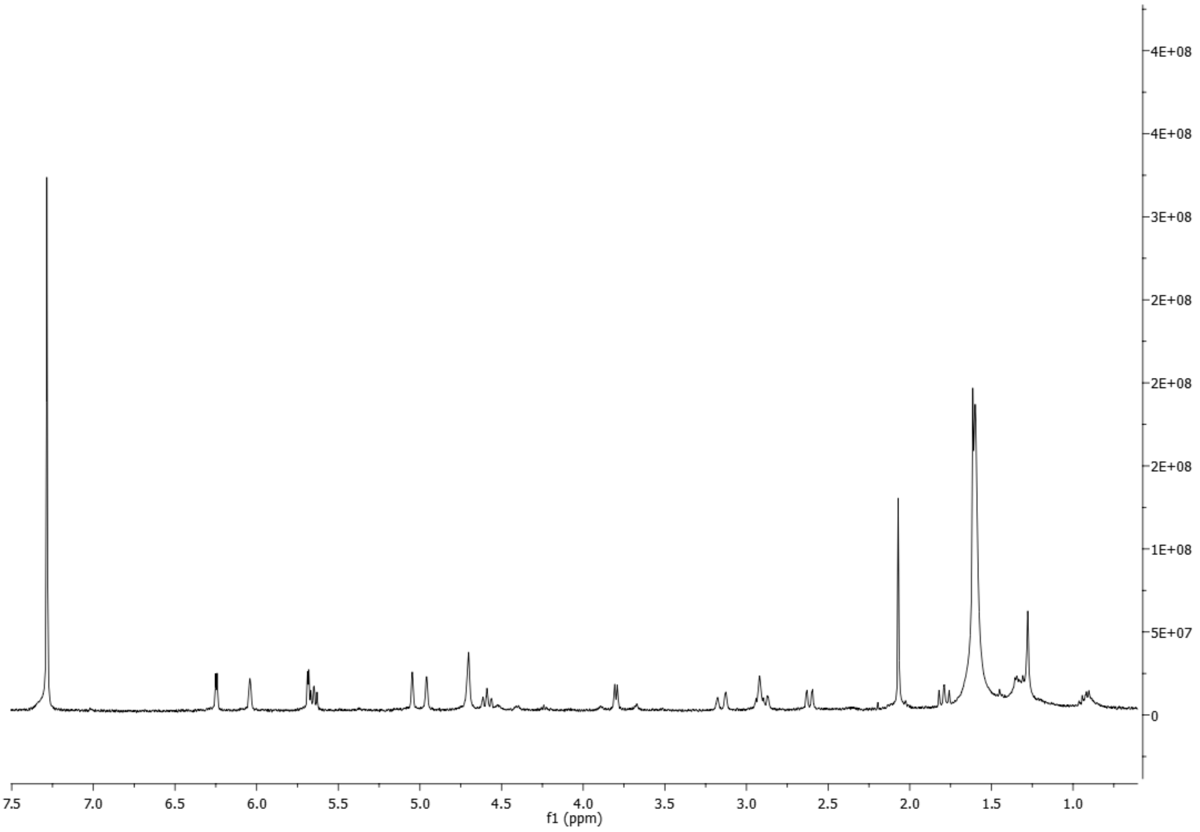

Figure S13. ^{1}H NMR spectrum of 6-acetoxy-10–β–hydroxyguaiantrienolide, **3** ($CDCl_3$, 400 MHz).

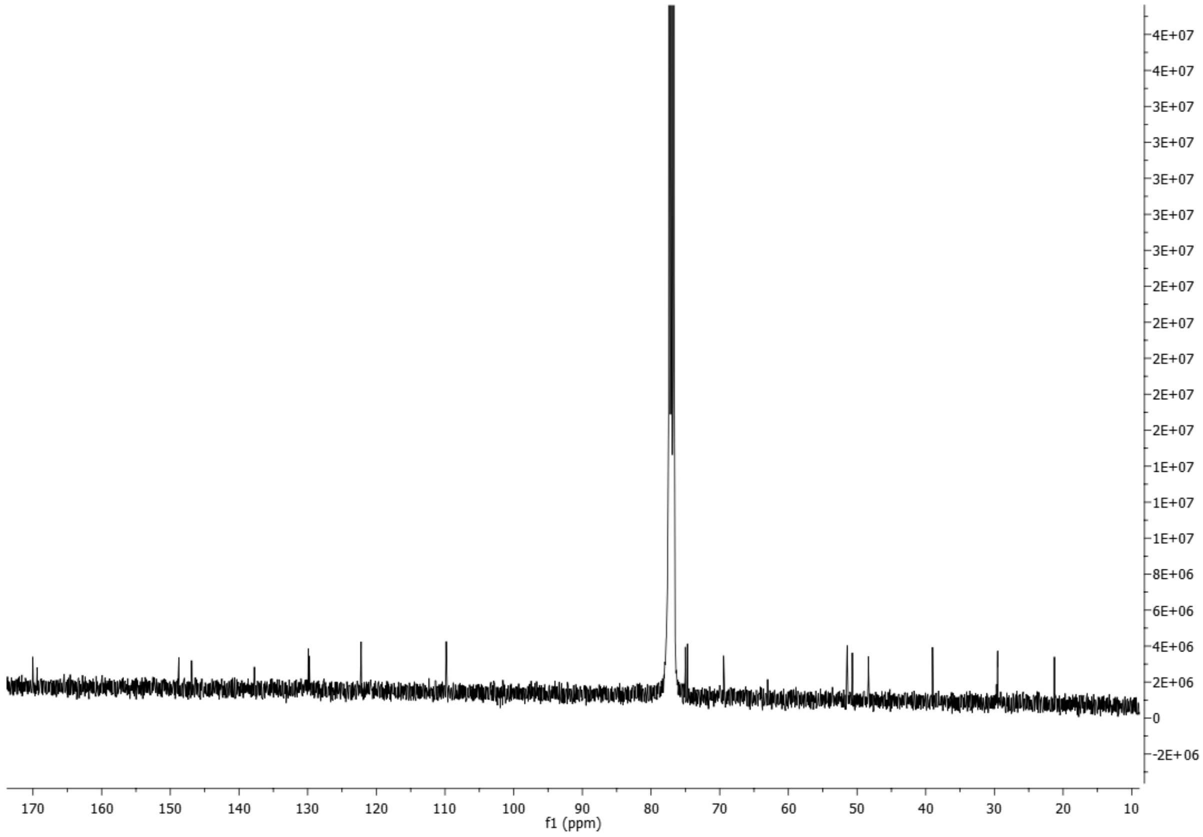

Figure S14. ^{13}C NMR spectrum of 6-acetoxy-10−β−hydroxyguaiantrienolide, **3** ($CDCl_3$, 100 MHz).

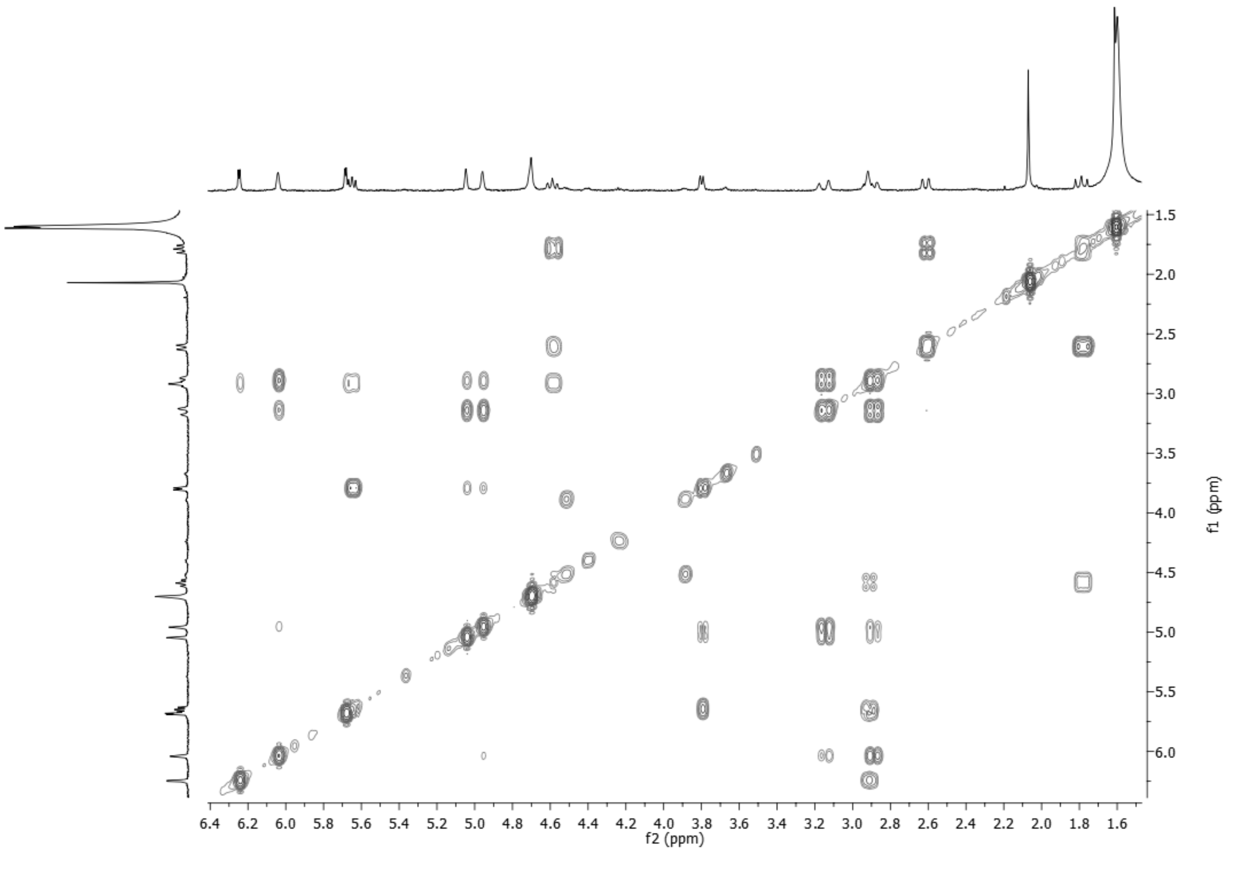

Figure S15. COSY spectrum of 6-acetoxy-10−β−hydroxyguaiantrienolide, **3** ($CDCl_3$, 400 MHz).

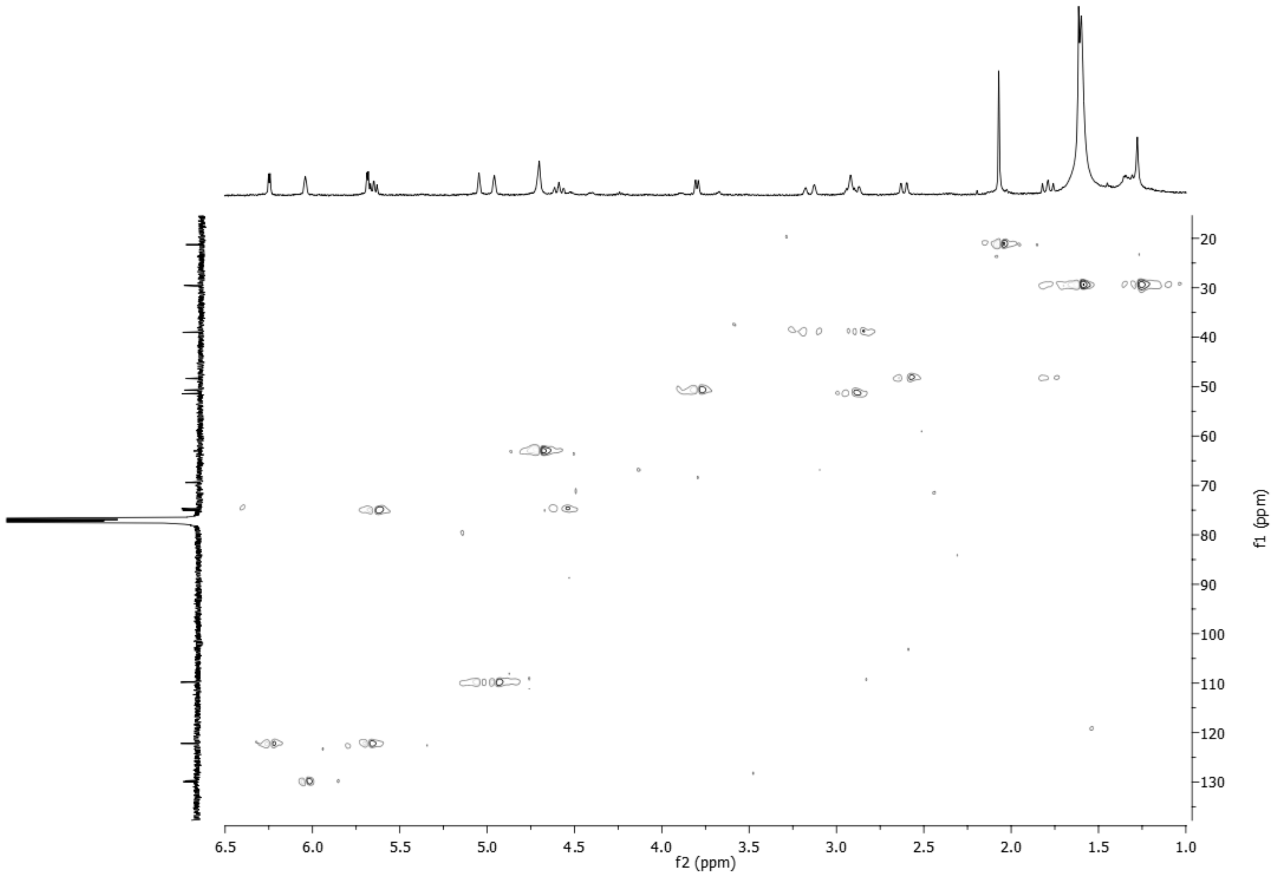

Figure S16. HSQC spectrum of 6-acetoxy-10–β–hydroxyguaiantrienolide, **3** ($CDCl_3$, 400/100 MHz).

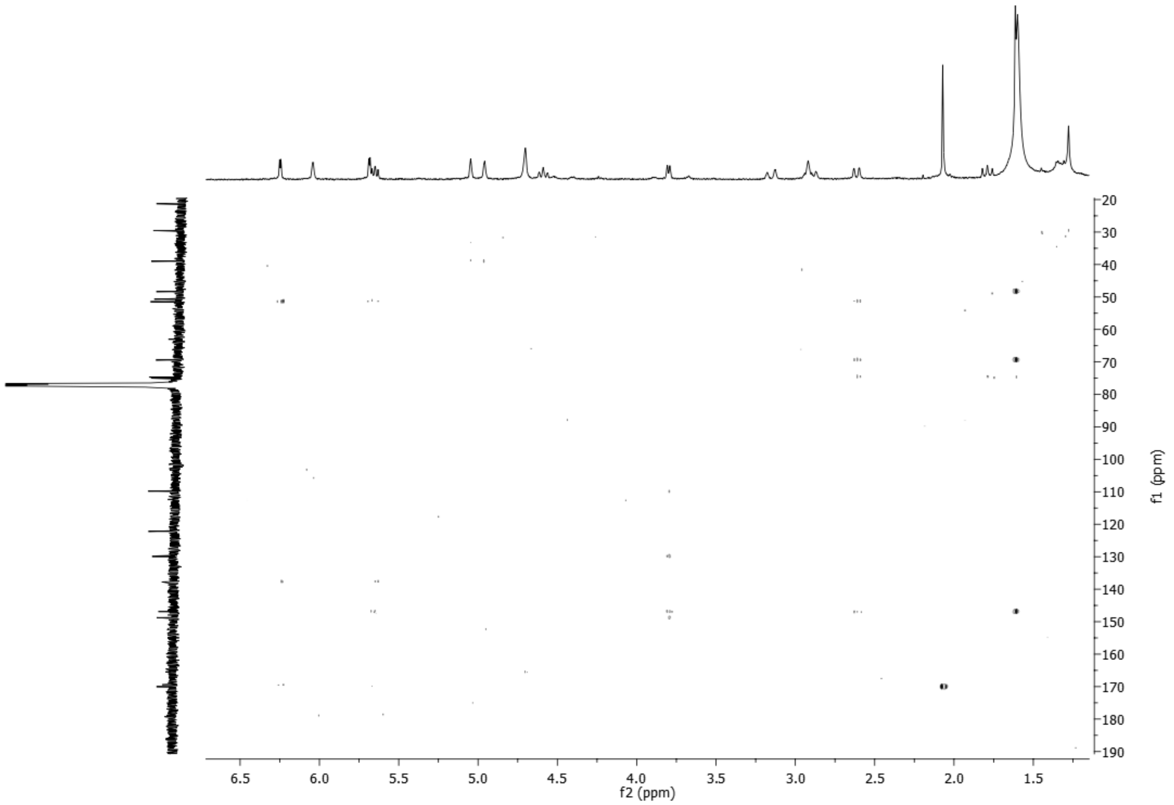

Figure S17. HMBC spectrum of 6-acetoxy-10–β–hydroxyguaiantrienolide, **3** ($CDCl_3$, 400/100 MHz).

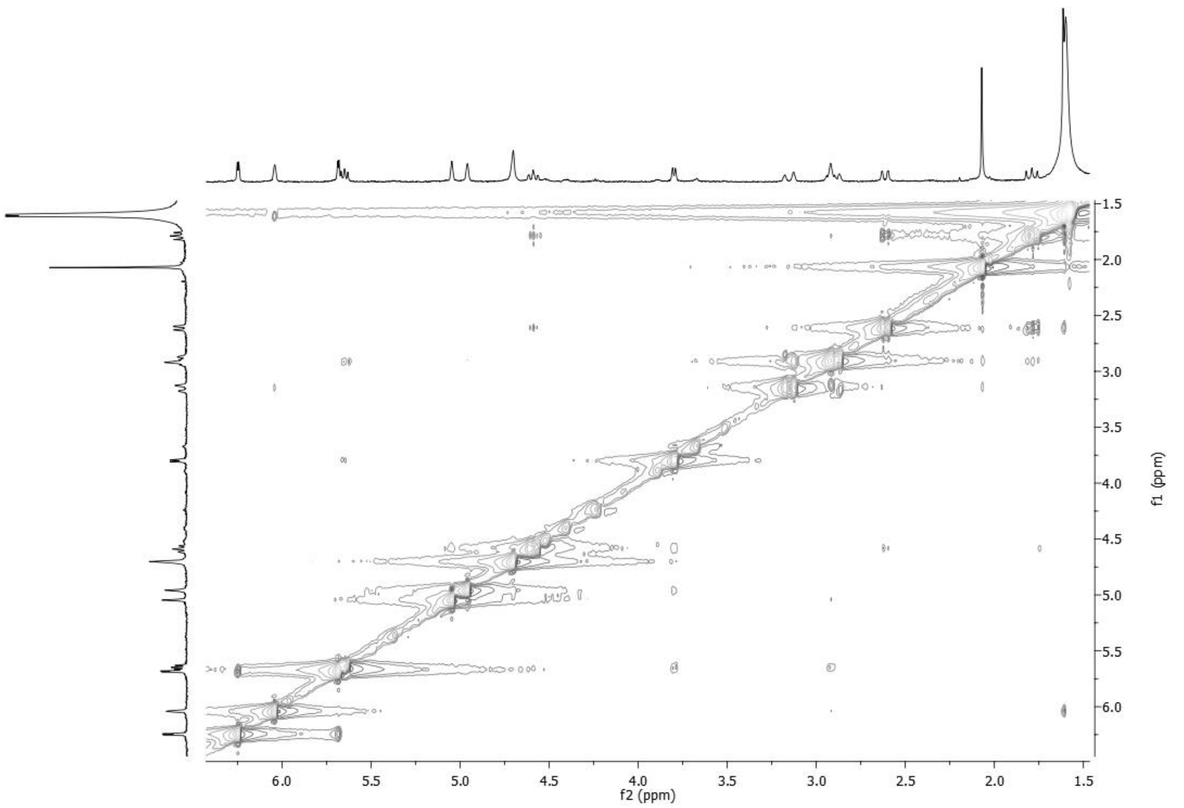

Figure S18. NOESY spectrum of 6-acetoxy-10–β–hydroxyguaiantrienolide, **3** ($CDCl_3$, 400 MHz).

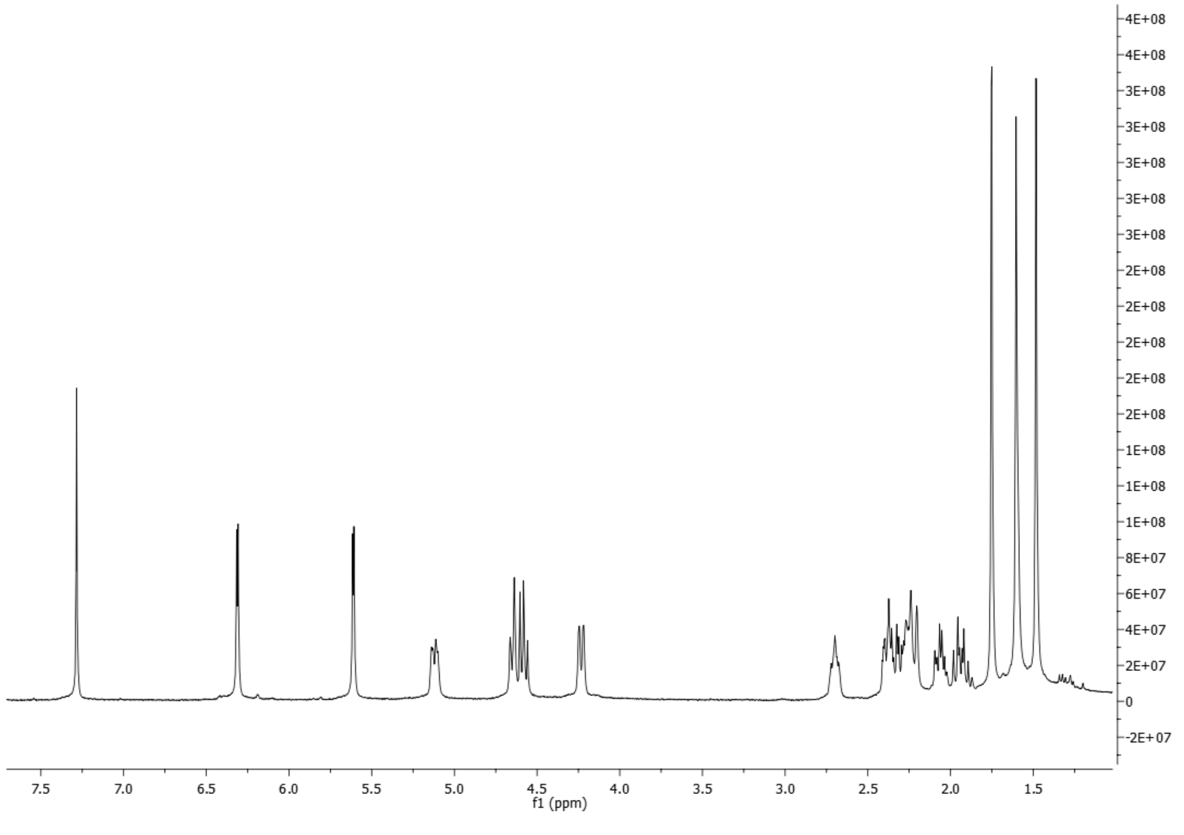

Figure S19: ^{1}H NMR spectrum of haagenolide, **4** ($CDCl_3$, 400 MHz).

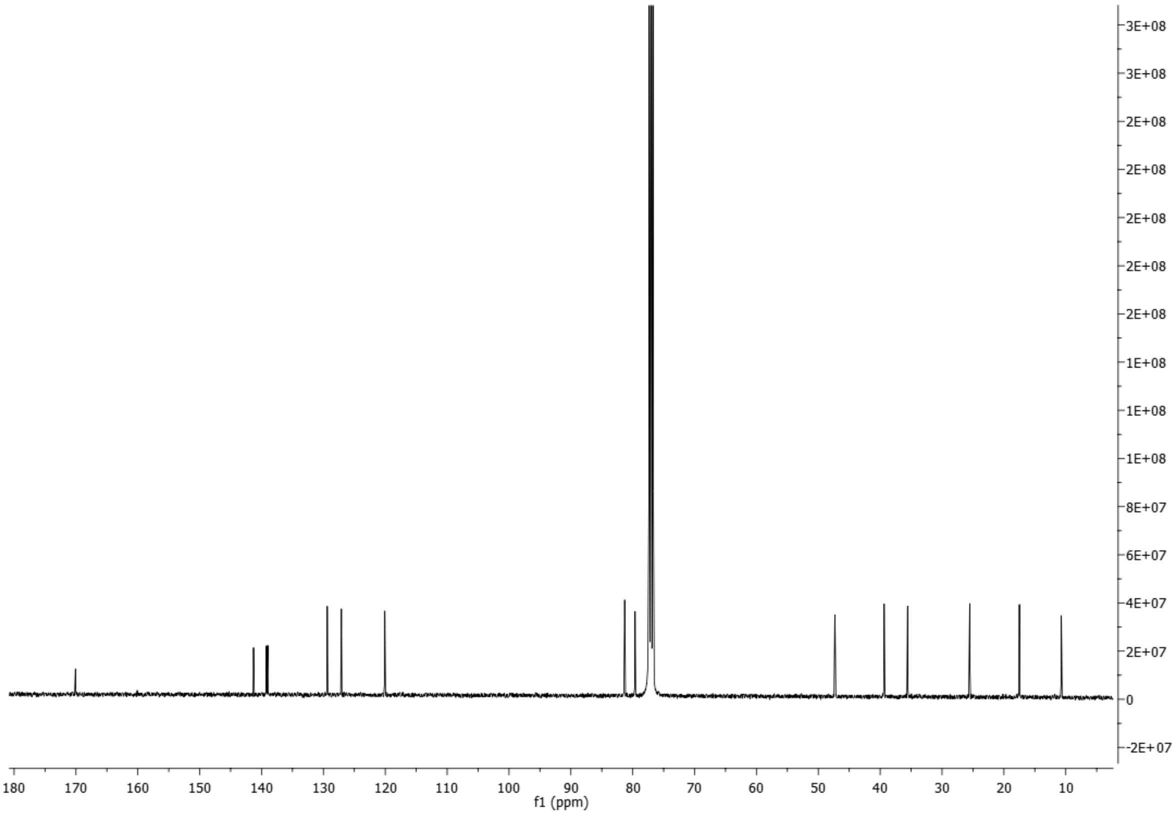

Figure S20. ^{13}C NMR spectrum of haagenolide, **4** (CDCl3, 100 MHz).

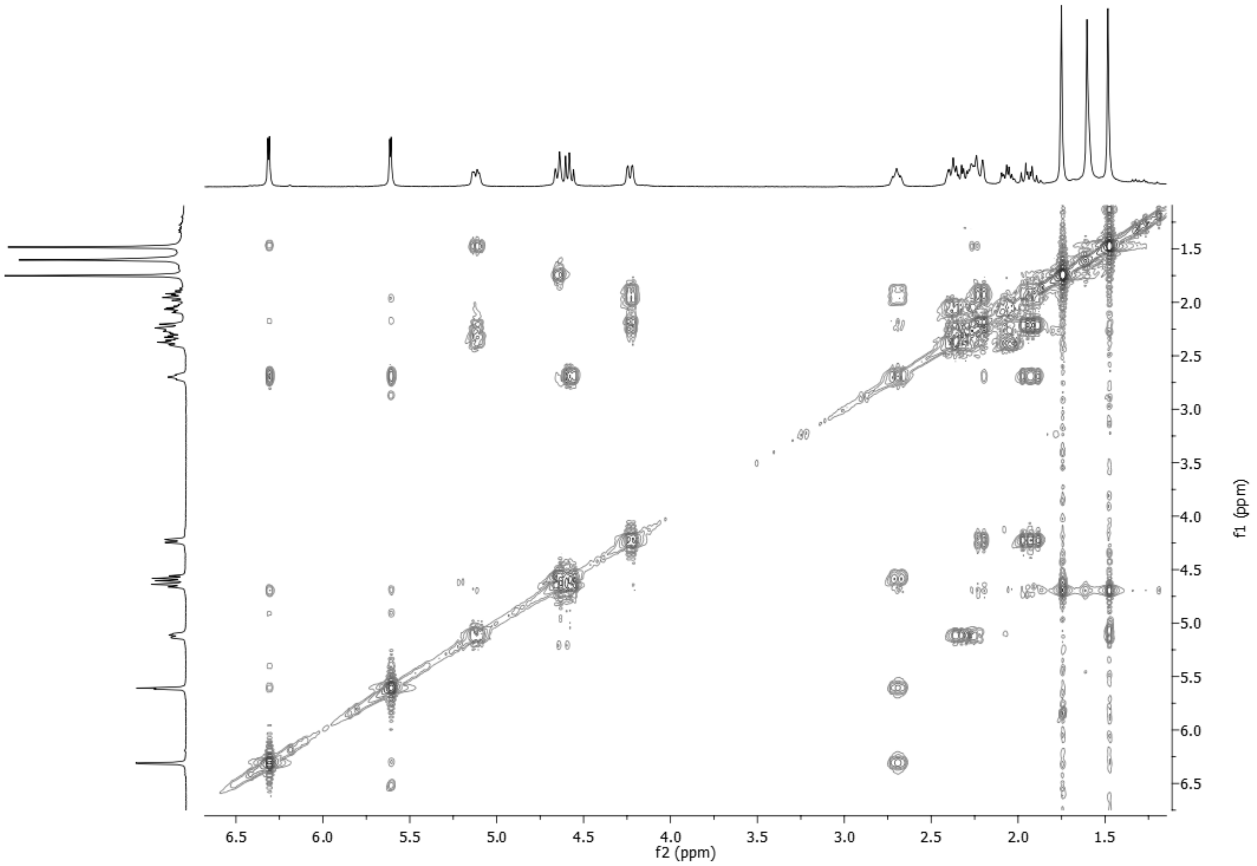

Figure S21. COSY spectrum of haagenolide, **4** ($CDCl_3$, 400 MHz).

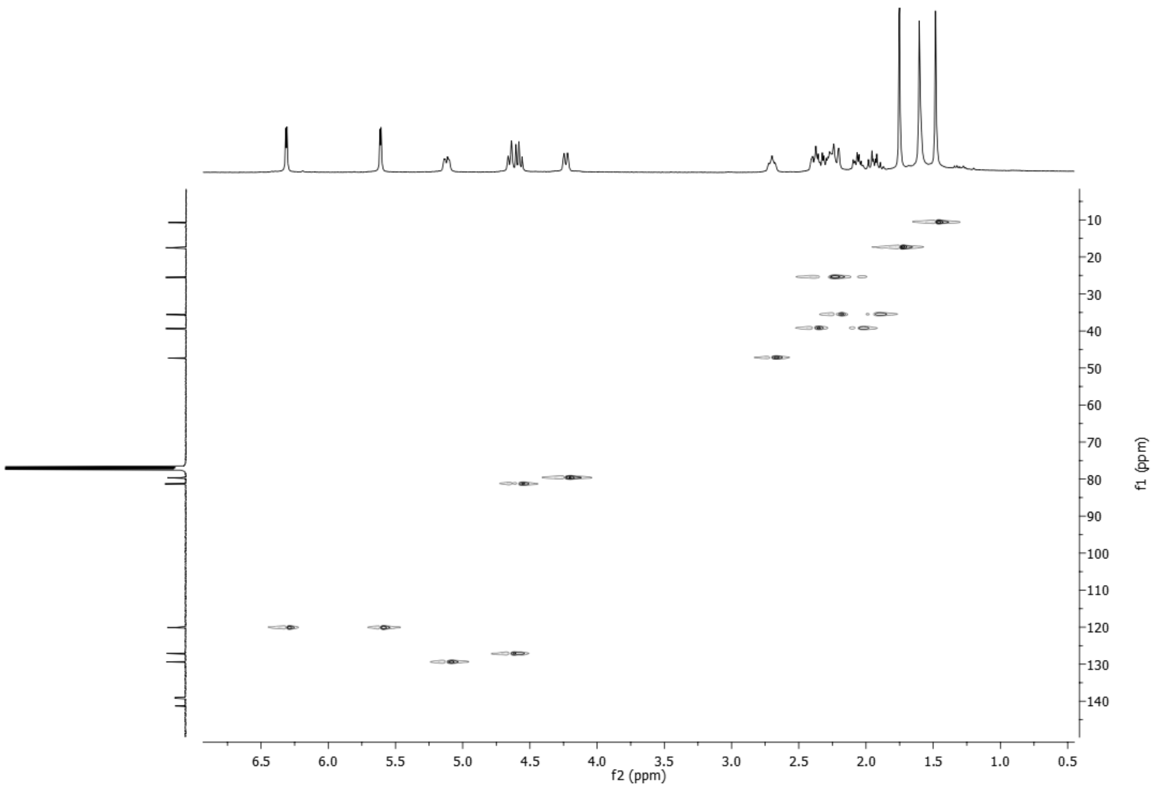

Figure S22. HSQC spectrum of haagenolide, **4** ($CDCl_3$, 400/100 MHz).

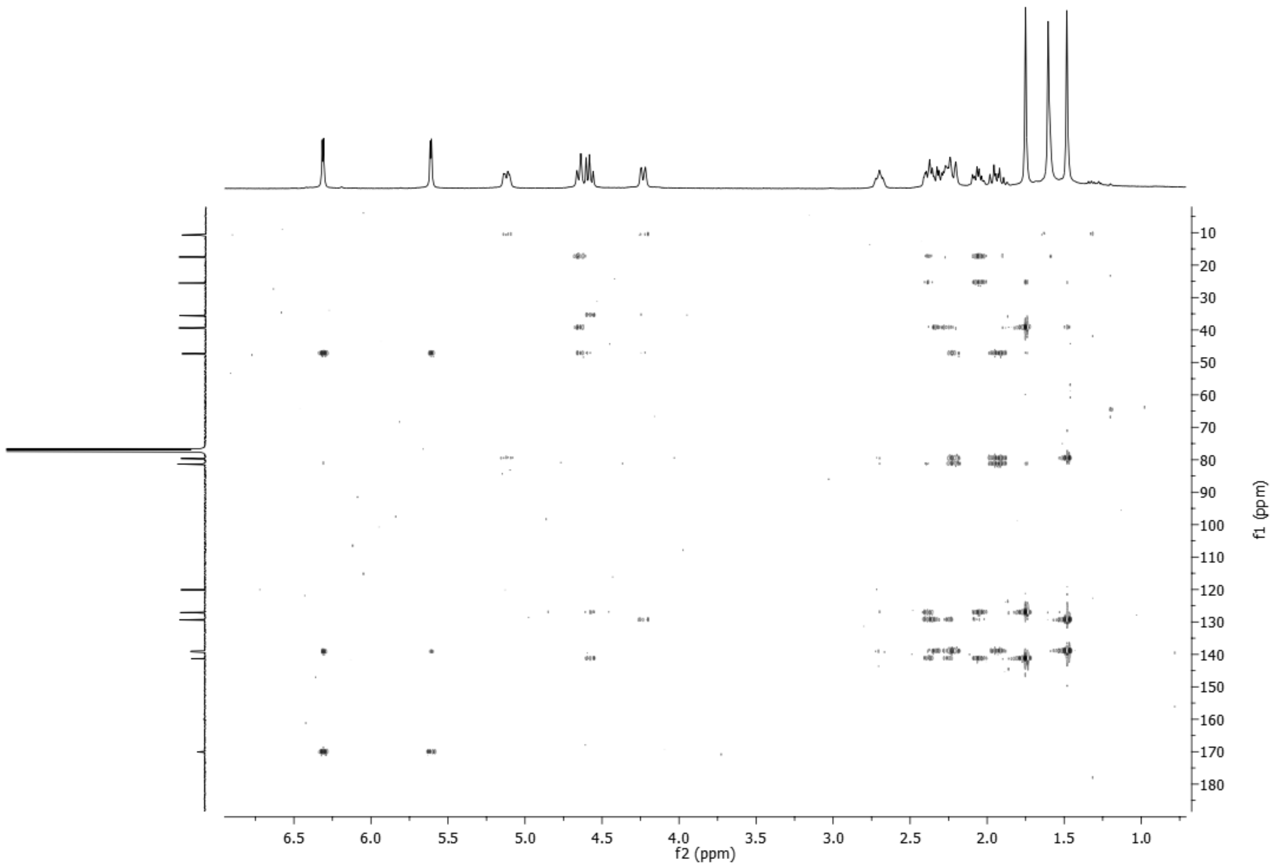

Figure S23. HMBC spectrum of haagenolide, **4** ($CDCl_3$, 400/100 MHz).

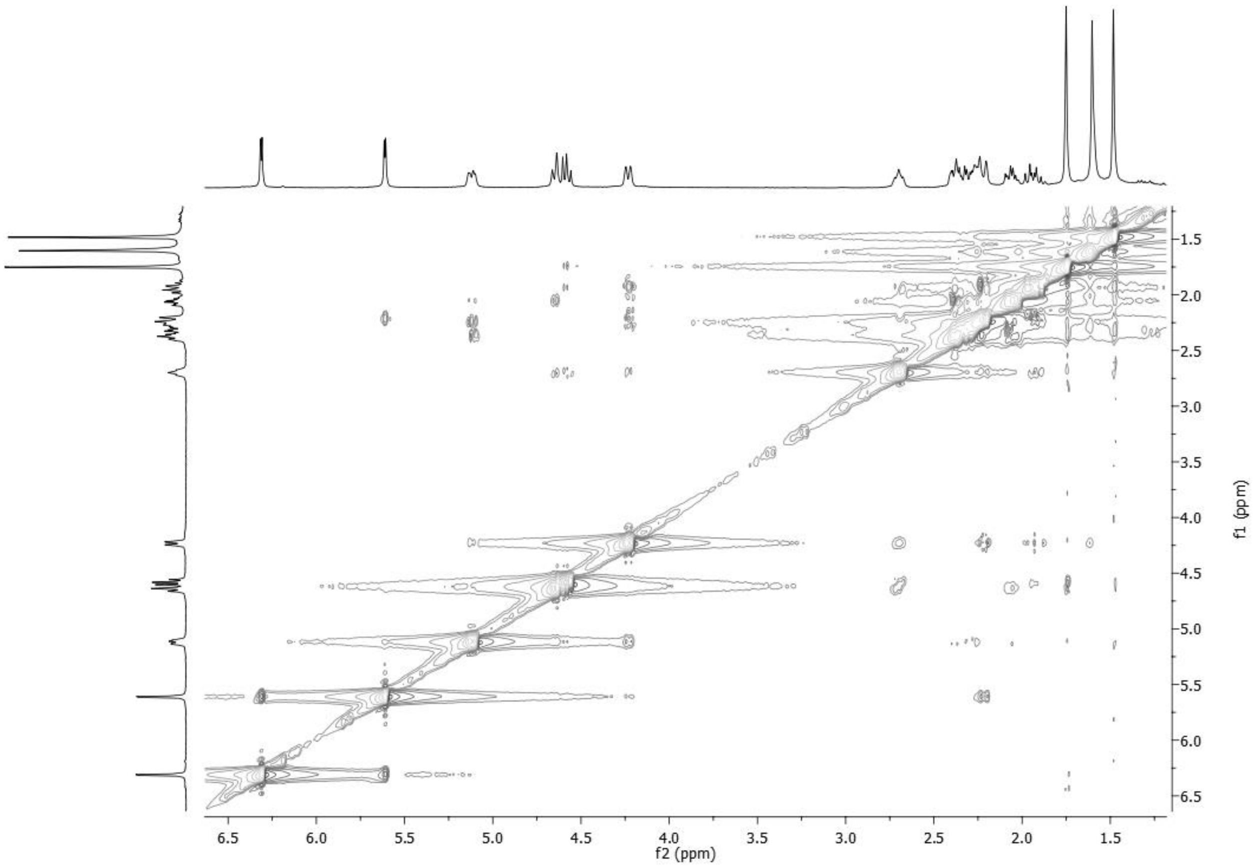

Figure S24. NOESY spectrum of haagenolide, **4** ($CDCl_3$, 400 MHz).

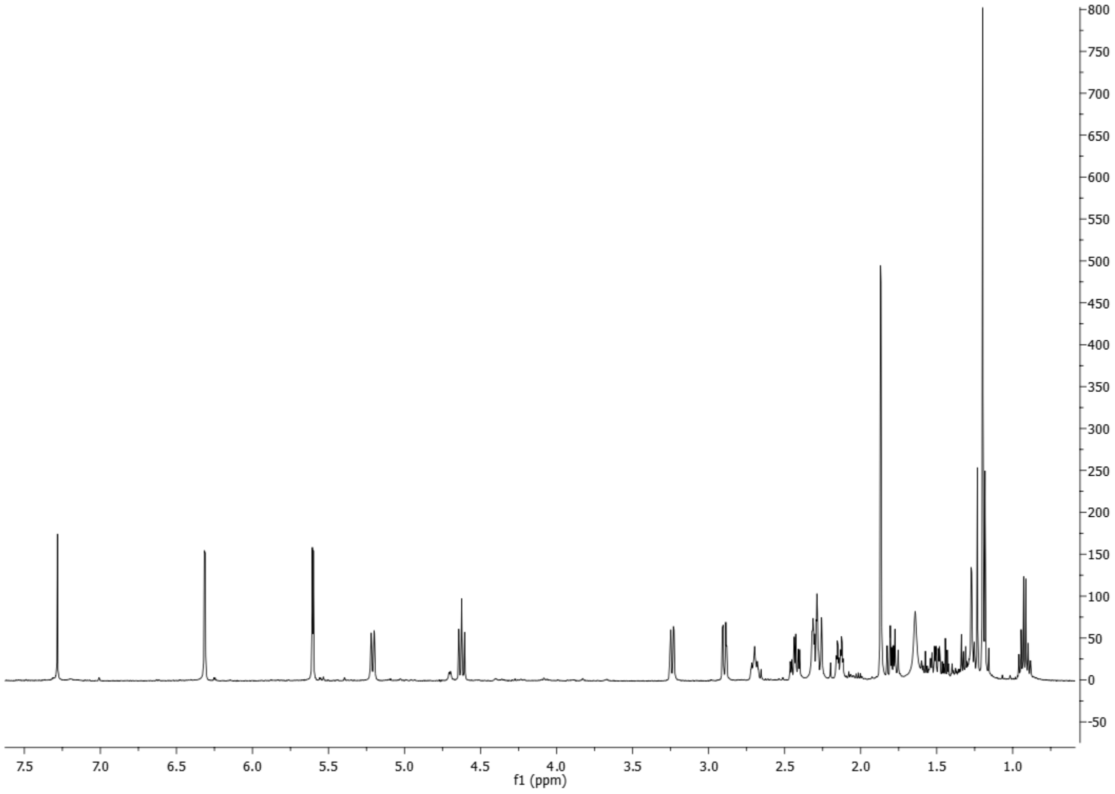

Figure S25: ^{1}H NMR spectrum of 1,10-epoxyhaagenolide, **5** ($CDCl_3$, 400 MHz).

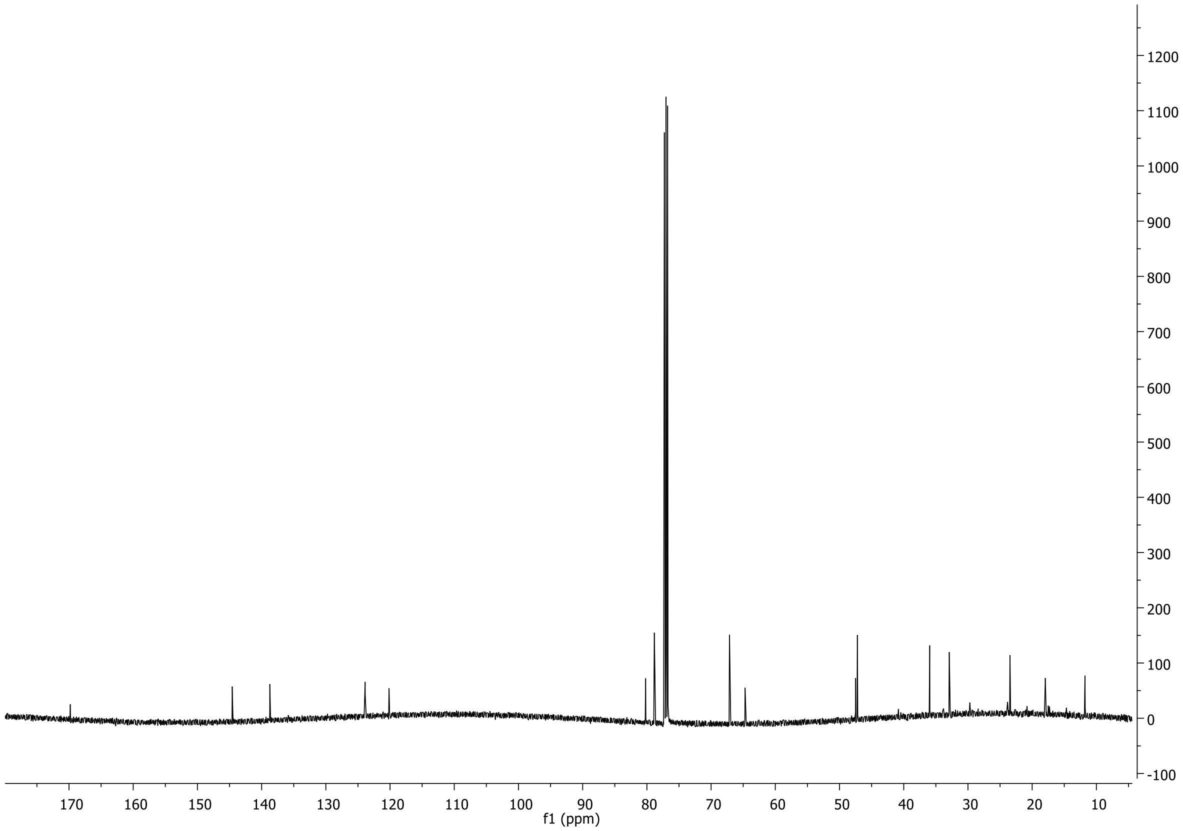

Figure S26. ^{13}C NMR spectrum 1,10-epoxyhaagenolide, **5** (CDCl3, 100 MHz).

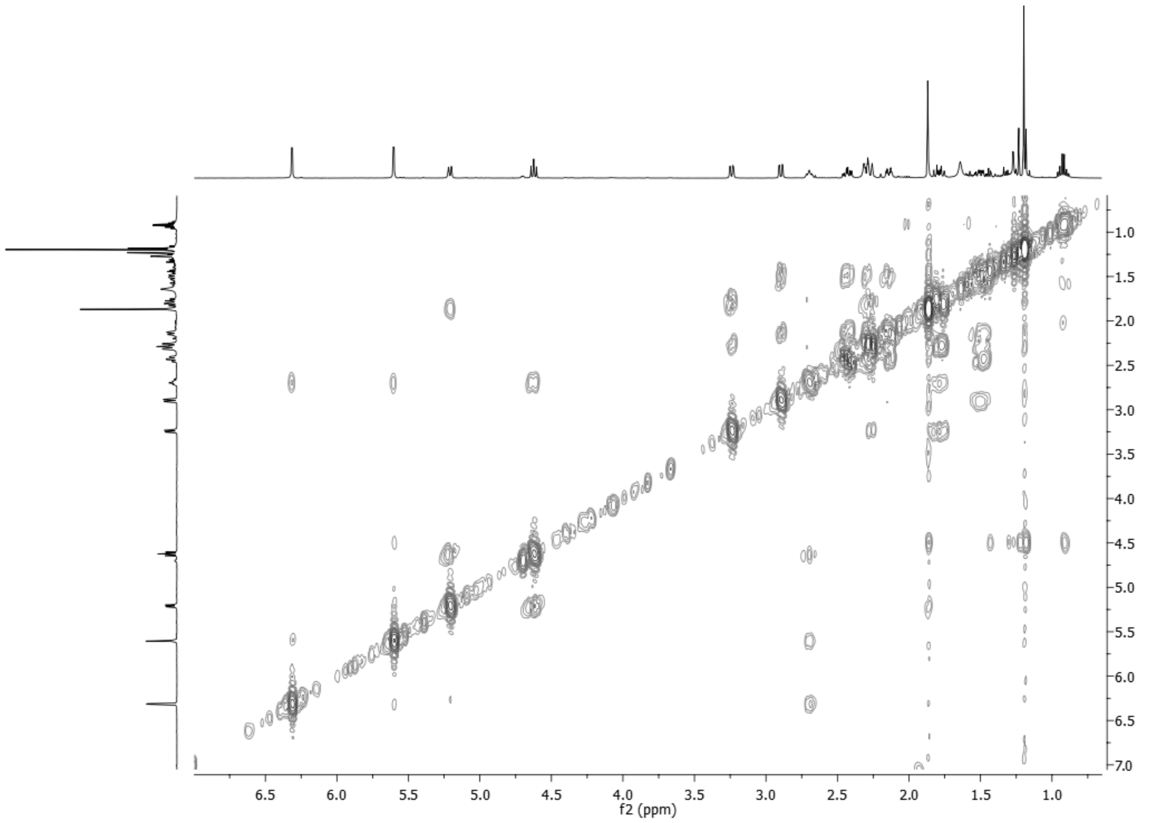

Figure S27. COSY spectrum of 1,10-epoxyhaagenolide, **5** **(**$CDCl_3$, 400 MHz).

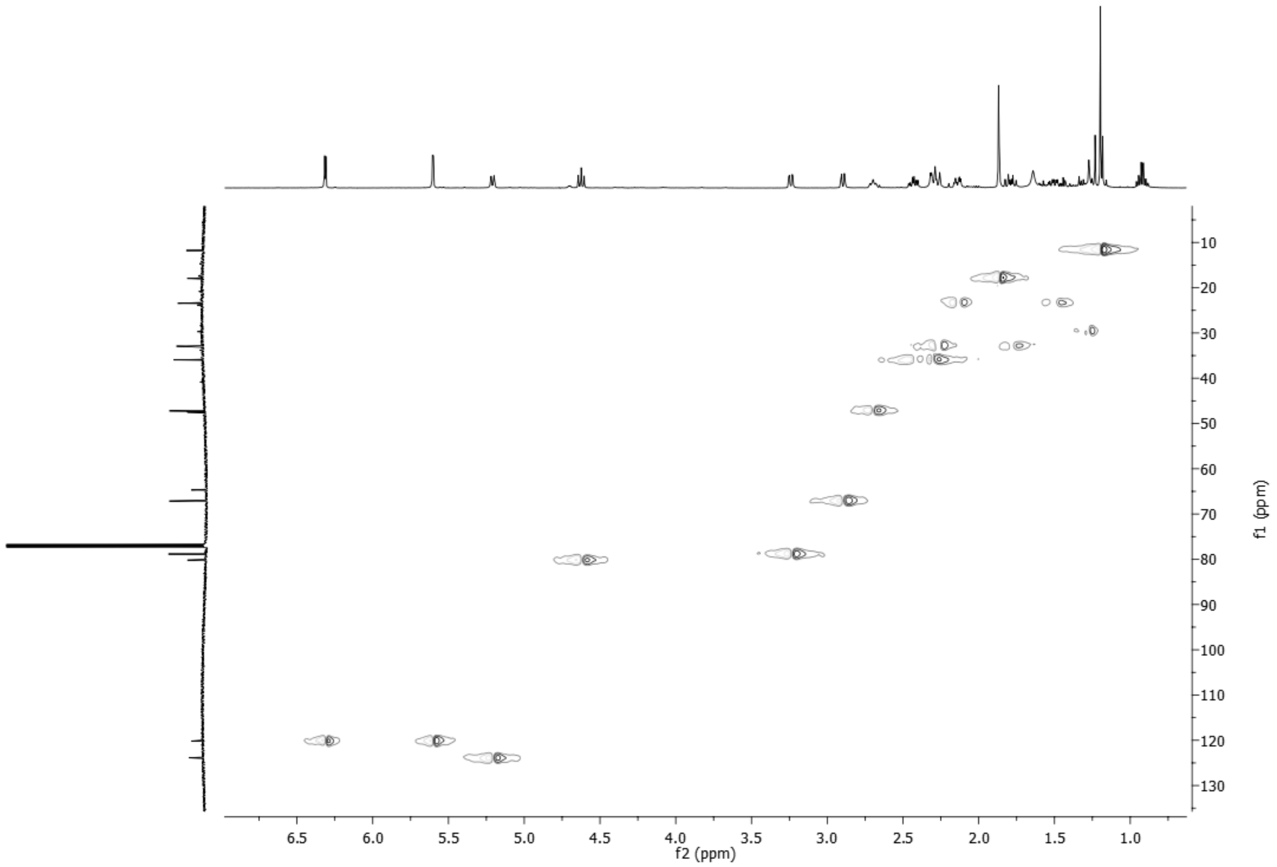

Figure S28. HSQC spectrum of 1,10-epoxyhaagenolide, **5** ($CDCl_3$, 400/100 MHz).

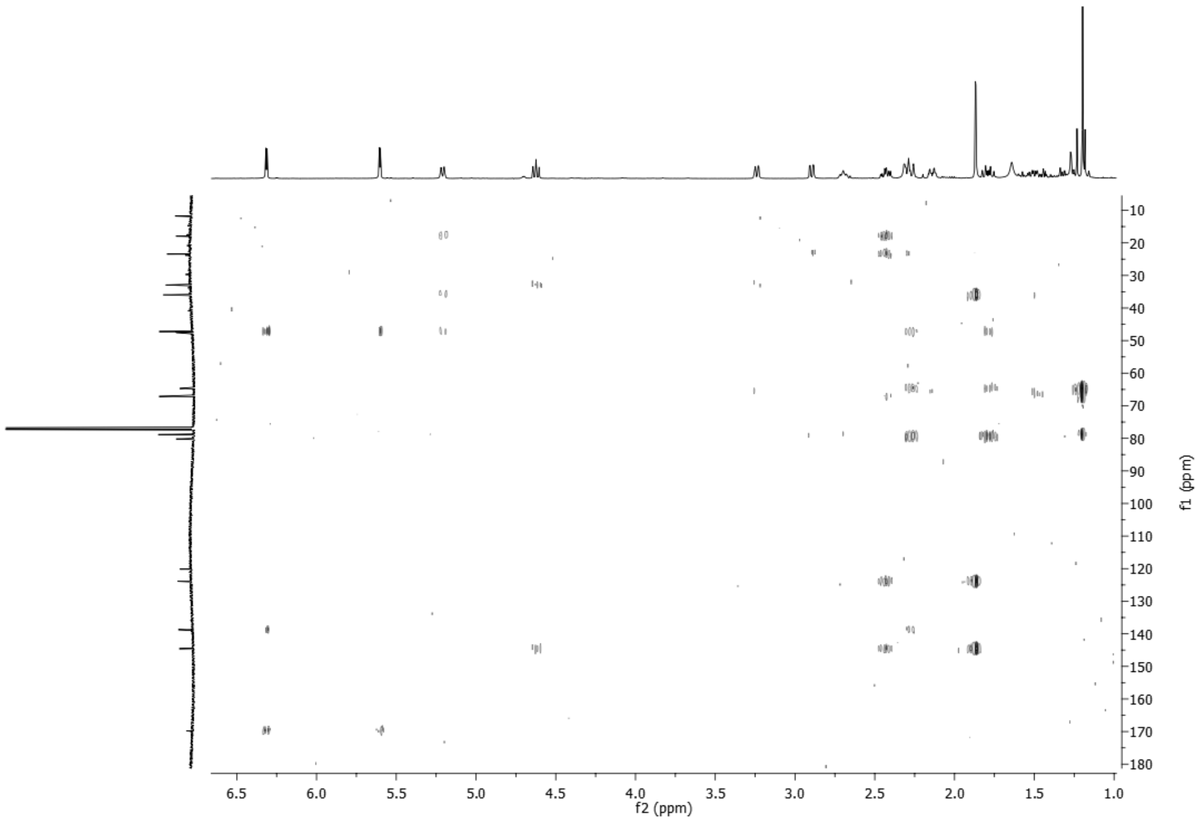

Figure S29. HMBC spectrum of 1,10-epoxyhaagenolide, **5** ($CDCl_3$, 400/100 MHz).

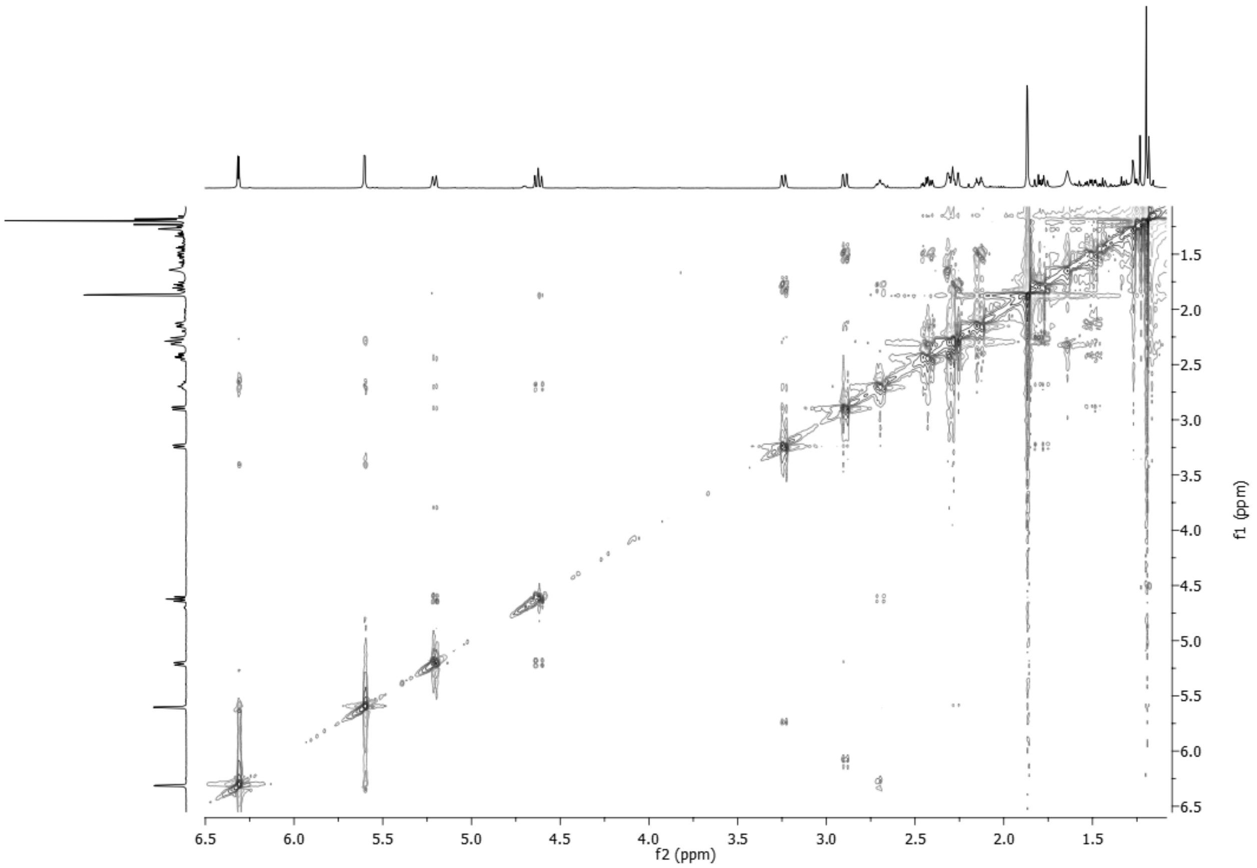

Figure S30. NOESY spectrum of 1,10-epoxyhaagenolide, **5** ($CDCl_3$, 400 MHz).

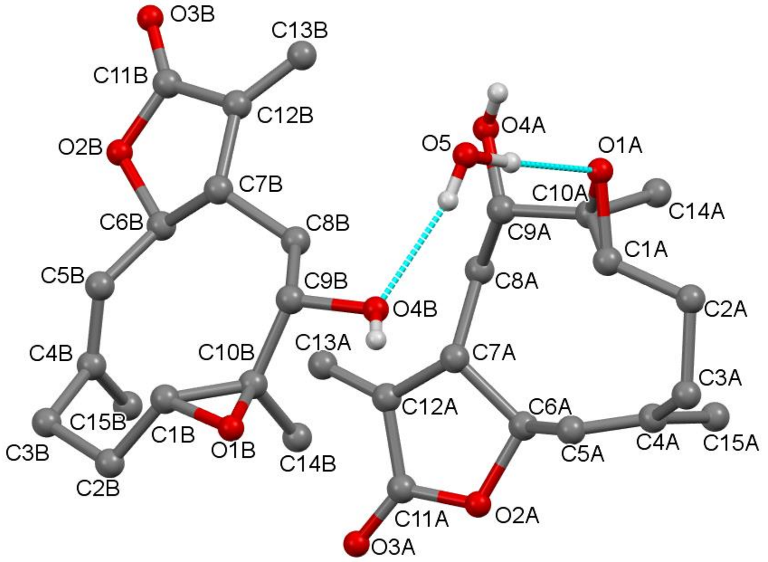

Figure S31. Perspective view of the crystallographic independent unit of **5** consisting of two independent molecules of **5** (named A and B) and one H_2O solvent molecule (only water and hydroxy H atoms are shown for clarity; hydrogen bonds are drawn as dashed cyan lines, ball and stick style). Geometric parameters of A and B molecules are very similar each to the other and clearly indicate the double bond C=C nature of C4–C5 and C12–C13 bonds, the presence of one carbonyl group (C11-O3) and one hydroxy group(O4-H) bonded at C9 (see Tables S2 and S3).

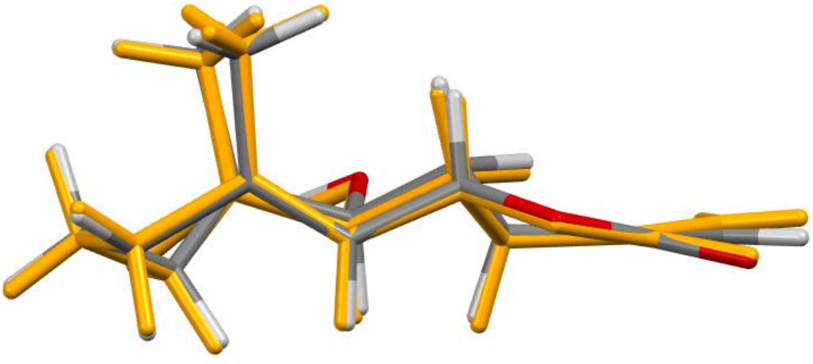

Figure S32. Perspective view of **5** with superimposition of the two independent molecules A (element colours) and B (orange). The two molecules have very similar geometric parameters and overlay at all. The very flat molecular shape observed is due to the chair–chair–chair conformation of the 10-membered ring and to the twist conformation of the 5-membered ring at the transjunction of the two rings.

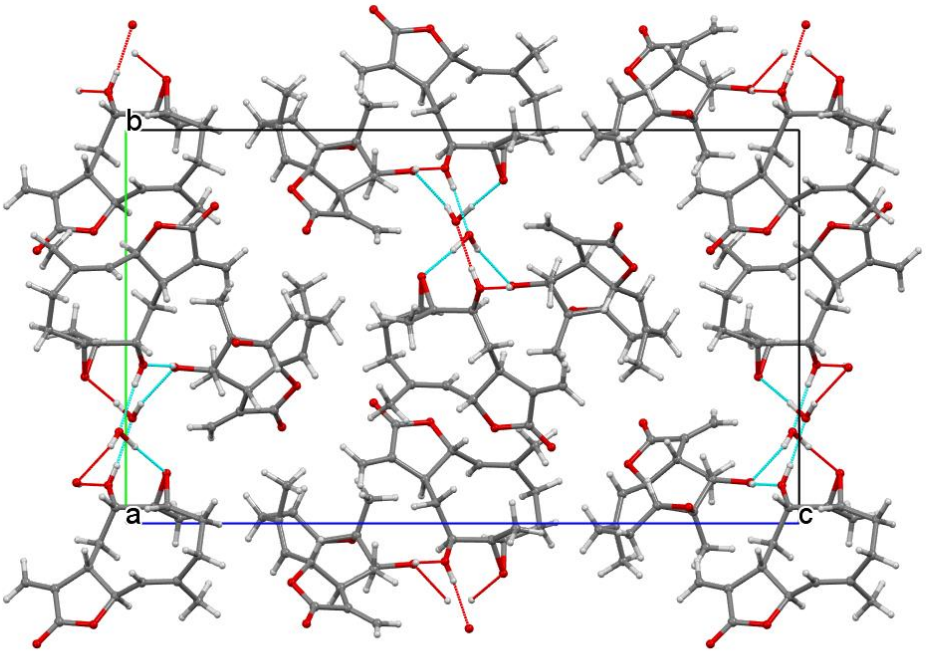

Figure S33. Crystal packing of **5** viewed down **a** axis. Molecules A and B of **5** and solvent water molecules are joined in a monodimensional H-bonding pattern through strong OH···O hydrogen bonds along **a** axis direction (hydrogen bonds are shown as cyan and red lines, ball-and-stick style).

Table S1. Crystal data and structure refinement parameters for **5**.

CCDC number	2076390
Empirical formula	C_{30} H_{42} O_9
Formula weight	546.63
Temperature	173(2) K
Wavelength	0.71073 Å

Crystal system	Orthorhombic
Space group	$P\ 2_1\ 2_1\ 2_1$
Unit cell dimensions	a = 7.6570(15)Å α= 90°
	b = 14.693(3)Å β = 90°
	c = 14.693(3)Å γ = 90°
Volume	2830.0(9)A^3
Z	4
Calculated density	1.283 Mg/m^3
Absorption coefficient	0.094 mm^{-1}
F(000)	1176
Crystal size	0.30 × 0.05 × 0.01 mm
Theta range for data collection	2.781° to 30.586°
Limiting indices	−10 ≤ h ≤ 10, −20 ≤ k ≤ 20, −35 ≤ l ≤ 35
Reflections collected / unique	78093 / 8560 [R(int) = 0.0502]
Refinement method	Full-matrix least-squares on F^2
Data / restraints / parameters	8560 / 0 / 368
Goodness-of-fit on F^2	1.077
Final R indices [I>2σ(I)]	R1 = 0.0455, wR2 = 0.0897
R indices (all data)	R1 = 0.0667, wR2 = 0.1003
Absolute structure parameter	0.1(2)
Extinction coefficient	n/a
Largest diff. peak and hole	0.261 and −0.252 $e\cdot A^{-3}$

Table S2. Selected bond lengths [Å] for (**5**).

C(1A)-O(1A)	1.452(3)	C(1B)-O(1B)	1.449(3)
C(10A)-O(1A)	1.471(3)	C(10B)-O(1B)	1.464(3)
C(6A)-O(2A)	1.473(2)	C(6B)-O(2B)	1.473(3)
C(11A)-O(2A)	1.357(3)	C(11B)-O(2B)	1.361(3)
C(11A)-O(3A)	1.202(3)	C(11B)-O(3B)	1.195(3)
C(9A)-O(4A)	1.423(3)	C(9B)-O(4B)	1.438(3)
C(4A)-C(5A)	1.334(3)	C(4B)-C(5B)	1.331(3)
C(12A)-C(13A)	1.320(3)	C(12B)-C(13B)	1.315(4)

Table S3. Hydrogen bonds for **5**. [Å and °]

D-H···A	d(D-H)	d(H...A)	d(D...A)	<(DHA)
C(1B)-H(1B)···O(3B)#1	1.00	2.58	3.374(3)	136.4
C(8B)-H(8B1)···O(5)	0.99	2.65	3.523(3)	146.9
O(4A)-H(4C)···O(5)#2	0.79(3)	1.89(3)	2.668(3)	167(3)
O(4B)-H(4D)···O(4A)#1	0.80(3)	2.02(3)	2.757(3)	154(3)
O(5)-H(5D)···O(1A)	0.96(4)	1.90(4)	2.861(3)	177(3)
O(5)-H(5E)···O(4B)	0.79(4)	2.10(4)	2.869(3)	164(4)

Symmetry transformations used to generate equivalent atoms: #1: x + 1, y, z; #2: x − 1/2, −y + 3/2, −z + 1
